# Trans10,cis12 conjugated linoleic acid inhibits proliferation and migration of ovarian cancer cells by inducing ER stress, autophagy, and modulation of Src

**DOI:** 10.1371/journal.pone.0189524

**Published:** 2018-01-11

**Authors:** Mian M. K. Shahzad, Mildred Felder, Kai Ludwig, Hannah R. Van Galder, Matthew L. Anderson, Jong Kim, Mark E. Cook, Arvinder K. Kapur, Manish S. Patankar

**Affiliations:** 1 Division of Gynecologic Oncology, Department of Obstetrics and Gynecology, University of Wisconsin School of Medicine and Public Health, Madison, Wisconsin, United States of America; 2 Department of Obstetrics and Gynecology Moffitt Cancer Center, Tampa, Florida, United States of America; 3 Division of Gynecologic Oncology, Department of Obstetrics and Gynecology, Dan L. Duncan Cancer Center, Baylor College of Medicine, Houston, Texas, United States of America; 4 Department of Animal Sciences, University of Wisconsin School-Madison, Madison, Wisconsin, United States of America; University of South Alabama Mitchell Cancer Institute, UNITED STATES

## Abstract

The goal of this study was to investigate the anti-cancer effects of Trans10,cis12 conjugated linoleic acid (t10,c12 CLA). MTT assays and QCM^™^ chemotaxis 96-wells were used to test the effect of t10,c12 CLA on the proliferation and migration and invasion of cancer cells. qPCR and Western Blotting were used to determine the expression of specific factors. RNA sequencing was conducted using the Illumina platform and apoptosis was measured using a flow cytometry assay. t10,c12 CLA (IC_50_, 7 μM) inhibited proliferation of ovarian cancer cell lines SKOV-3 and A2780. c9,t11 CLA did not attenuate the proliferation of these cells. Transcription of 165 genes was significantly repressed and 28 genes were elevated. Genes related to ER stress, ATF4, CHOP, and GADD34 were overexpressed whereas EDEM2 and Hsp90, genes required for proteasomal degradation of misfolded proteins, were downregulated upon treatment. While apoptosis was not detected, t10,c12 CLA treatment led to 9-fold increase in autophagolysosomes and higher levels of LC3-II. G1 cell cycle arrest in treated cells was correlated with phosphorylation of GSK3β and loss of β-catenin. microRNA miR184 and miR215 were upregulated. miR184 likely contributed to G1 arrest by downregulating E2F1. miR215 upregulation was correlated with increased expression of p27/Kip-1. t10,c12 CLA—mediated inhibition of invasion and migration correlated with decreased expression of PTP1b and decreased Src activation by inhibiting phosphorylation at Tyr^416^. Due to its ability to inhibit proliferation and migration, t10,c12 CLA should be considered for treatment of ovarian cancer.

## Introduction

Trans10:cis12 Conjugated Linoleic Acid (t10,c12 CLA), an 18-carbon fatty acid belongs to a family of 28 isomers occurring naturally in dairy products and red meat [1, 2]. t10,c12 CLA and cis9:trans11 CLA (c9,t11 CLA) are the most abundant isomers that in in vitro and in vivo studies suppress proliferation of breast, colon, stomach, prostate, colorectal, and hepatic cancer cells [3–6].

In cancer cells, t10,c12 and c9,t11 CLA isomers induce apoptosis and cell cycle arrest [7, 8]. Mechanistic studies have linked the anti-cancer effects of these two CLA isomers to their ability to alter fatty acid composition, inhibit Cox-2 expression, induce p53, p27, and p21 proteins, suppress Her-2 and Bcl-2, and modulate the phosphorylation and activation of ErbB3, Akt and other key signaling molecules [8–13]. t10,c12 CLA induces apoptosis in the p53-mutant mouse mammary cancer cell line, TM4t, by perturbing homeostasis in the endoplasmic reticulum (ER) via oxidative stress and lipid peroxidation [7]. In addition to ER stress, t10-c12 CLA-induced apoptosis in the TM4t cells is also a result of G-protein coupled receptor (GPCR)-mediated activation of AMP-activated protein kinase [14]. Collectively, a survey of the literature indicates that (a) the t10,c12 and c9,t11 CLA isomers produce a gradation of anti-cancer effects in different cancer models, and (b) the inhibition of tumor cell proliferation is a result of modulation of multiple cell signaling pathways. The complexity of the molecular responses in the CLA treated cancer cells suggests that clear delineation of the molecular mechanisms behind the anti-cancer effects of these fatty acids will require the extensive use of “omics” strategies conducted in a cancer cell-type specific manner.

Serous epithelial ovarian cancer is the sixth most common cancer in women and despite advances in surgical and chemotherapeutic approaches is the leading cause of female mortality occurring due to gynecologic malignancies [15]. Therefore, there is an acute need to identify novel therapeutic approaches to prevent and treat ovarian cancer.

To the best of our knowledge, a systematic study on the effect of t10,c12 or c9,t11 CLA on ovarian cancer cells has not been conducted. Here, we demonstrate that t10,c12 CLA is a potent inhibitor of proliferation, invasion, and migration of ovarian cancer cells. Global gene microarray and microRNA sequencing analysis followed by targeted molecular experiments have led us to identify key molecular events that allow t10,c12 CLA to potently inhibit the proliferation and migration of ovarian cancer cells. Our results indicate that t10,c12 CLA should be considered as an important modality in the treatment of ovarian cancer.

## Materials and methods

### Cell culture and conditions

SKOV3 and A2780 cells were acquired from ATCC and maintained in RPMI 1640 supplemented with 15% fetal bovine serum (FBS) and 0.1% gentamicin sulfate (Gemini Bio-products, Woodland, CA) in 5% CO_2_/95% air at 37°C.

### In vitro CLA treatment

SKOV3 and A2780 were plated (75X10^4^/10 cm plate) and serum starved overnight. Cells were washed with phosphate buffered saline (PBS) and treated with 7 μM t10,c12 CLA for 48 to 72 h in serum free media (substituted with Mito+, BD Biosciences, CA). Cells were harvested using 0.05% trypsin in Hanks balanced salt solution (HBSS) containing 25 mM HEPES, resuspended in RPMI 1640 containing 10% fetal bovine serum (FBS) and washed twice with PBS.

### Apoptosis and cell cycle assay

After treatment for 48 and 72 h with CLA isomers (at IC_50_ doses, 7 μM), SKOV-3 and A2780 cells were harvested, washed with PBS and either fixed (cell cycle assay) in 75% ethanol, washed with PBS and stained with propidium iodide or freshly incubated (apoptosis assay) with 5 μL of Annexin V-FITC and PI (BD Pharmingen, San Diego, CA) for 15 minutes. Flow cytometry was then performed to analyze the samples for both apoptosis and cell cycle status as described earlier [16].

### Proliferation assays

SKOV-3 and A2780 cells were plated (1.5X10^3^ cells) in 96-well culture plates in serum containing conditions for 24 hours. Cells were maintained overnight in serum free media supplemented with Mito^+^ serum extender (BD Biosciences). Media was replaced with serum free media containing different concentration of CLA isomers. Treatment was stopped at 24-, 48-, 72-, and 96-hours and 0.15% MTT added to each well followed by incubation at 37°C for 2 h. Media was removed, DMSO (100 μL; Sigma-Aldrich) was added and absorbance at 570 nm was measured using Spectramax microplate reader (Molecular Devices, Sunnyvale, CA). Each sample was analyzed in quadruplicate.

### Invasion and migration assay

After treatment with *t10*,*c12* CLA for 48h hours, cells (1.0X10^5^) were added to appropriate wells in the QCM^™^ chemotaxis 96-well migration assay (Chemicon International) plate with or without 10% FBS (for chemotaxis) to the feeder tray, according to the manufacturers’ recommendations and previously described [17, 18]. All experiments were done in 5 replicates. Cells were then incubated in 37°C for 24 h. Media from upper inserts was gently discarded and migration tray placed into new 96-well feeder tray containing 150 μL of pre-warmed cell detachment solution and incubated for 30 minutes at 37°C. Next, cells were lysed in buffer containing CyQuant GR Dye (Molecular Probes, Eugene, OR) and incubated at room temperature for 15 minutes. Mixture was then transferred to a new 96-well plate suitable for fluorescence measurement (Corning Incorporated, Corning, NY). Plate was then read with fluorescence plate reader (Molecular Devices, Sunnyvale, CA) using 480/520 nm filter set. Similarly for invasion assay we used QCM^™^96-well cell invasion assay (Chemicon International). The protocol for the assay is same as for the migration assay. This plate contains 96 inserts; each insert contains an 8 μm pore size polycarbonate membrane coated with a thin layer of ECMatrixTM. The ECM layer occludes the membrane pores, blocking non-invasive cells from migrating through. Invasive cells, on the other hand, migrate through the ECM layer and cling to the bottom of the polycarbonate membrane. Invaded cells on the bottom of the insert membrane are dissociated from the membrane when incubated with Cell Detachment Buffer and subsequently lysed and detected by CyQuant GR dye by measuring the fluorescence by a plate reader (Molecular Devices, Sunnyvale, CA) using 480/520 nm filter set.

### Western blot analysis

Briefly, lysates from cultured cells were prepared with modified RIPA buffer and protein concentrations were determined with a BCA Protein Assay Reagent kit (Pierce Biotechnology, Rockford, IL). Lysates were separated using 10% sodium dodecyl sulfate–polyacrylamide gels. Proteins were transferred to PVDF membrane, blocked with 5% milk and incubated at 4°C with primary antibody overnight: Src (anti-rabbit; cell signaling), Caspase3, MAPK, GSK3β, P27, β-Catenin (against Src [Abcam, Cambridge, MA]), after washing with TBST, the membranes were incubated with 1 μg/mL horseradish peroxidase (HRP)–conjugated horse anti-rabbit IgG (Amersham, Piscataway, NJ). Bands were detected using chemiluminescence detection.

### Microarray analysis

The experiment was run in triplicate. Cells from the same passage were labeled at the same time with t10,c12 CLA. After treatment, the cells were harvested and RNA was extracted at the same time using identical conditions and the same reagents. The samples were checked for the quality of the RNA and for microarray analysis. Total RNA was isolated from t10,c12 CLA-treated A2780 cells using mirVana isolation kit (Ambion Inc., Austin, TX) according to the manufacturer’s instructions and quantified by a NanoDrop ND-1000 spectrophotometer. For quality control, 1 μl of the diluted RNA (dilution was the working concentration of the Agilent RNA 6000 nano kit) was run on the Nano chip using an Agilent 2100 electrophoresis Bioanalyzer to obtain electropherogram profile. For each biological replicate 50 ng of RNA was labeled using Illumina TotalPrep RNA Amplification kit from Ambion. Then 1.5 μg of cRNA was mixed with hybridization controls and hybridized on Illumina’s HT12V4 expression bead chip array for 16 h in a hybridization oven with a rocking platform at 58°C. The Chip was then washed and stained with streptavidin-Cy3 and scanned using Illumina Bead Array reader. The microarray data was normalized using an endogenous reference gene. By conducting the sequencing at the same time, the inter-chip variability was circumvented. The microarray data was analyzed using Genesifter software from Geospiza. The data was log transformed and linear modeling of the transformed data was performed with Benjamini and Hoechberg correction. The expression levels with 1.5 fold difference and false discovery rate below 5% was considered as significantly differentially regulated.

### Transmission electron microscopy

Following dialysis against 0.125 M ammonium acetate, 2.6 mM ammonium carbonate, 0.26 mM EDTA, pH 7.4, the samples were negatively stained with 2% sodium phosphotungstate, pH 7.2 and placed on Formvar/Carbon coated 200 mesh nickel grid support films (Ted Pella, Inc.). The autophagosome were visualized (magnification of 50,000) using a Zeiss 910 transmission electron microscope. The photographs obtained were enhanced and autophagosomes per field were counted using Adobe Imageready CS2 software.

### RNA extraction and cDNA

After appropriate treatment, cells were washed with PBS twice. Total RNA was isolated using mirVana miRNA Isolation kit (Ambion, Inc., Austin, TX). RNA quality was confirmed and only RNA with greater than 1.5 OD ratio (260/280) was used to make the complementary DNA (cDNA). The cDNA was generated with 1.0 μg of RNA using RT^2^ miRNA First Strand Kit (Qiagen) as previously described [19].

### MicroRNA sequencing

Total RNA was isolated using mirVana miRNA Isolation kit (Ambion, Inc., Austin, TX). To assess RNA quality, each specimen was assessed using a 2100 Bioanalyzer (Agilent, Inc., Santa Clara, CA). Small RNA libraries were prepared for each sample using version 1.5 of Illumina’s small RNA prep kit to process 1 μg of total RNA according to the manufacturer’s protocol (Illumina, Inc., San Diego, CA). Purified cDNA was quantified with the Quant-iT PicoGreen dsDNA kit (Molecular Probes, Eugene, OR) and diluted to 10 nM for sequencing on Illumina’s Solexa 2G Genome Analyzer by the Institute of Molecular Design Sequencing Center at the University of Houston (http://imd.uh.edu). All read counts were normalized prior to statistical analysis as described previously [20].

### RNA analysis and mapping

Sequence reads obtained from individual runs were filtered using the Illumina chips and no-call filters. Any transcript up to 36 nucleotides (nt) in length was considered for miRNA screening, compensating for the 3’ addition of the Illumina adaptor initially ligated to small RNAs prior to their cloning. Reads were removed from further analysis if copy number for a transcript was < 4, length was < 10 nt or if a transcript contained >10 consecutively repeated bases. Transcripts originating from E. coli were also removed from our pool of sequence reads using WU Blast. Remaining sequence reads were then mapped to known human miRNA hairpins using mirBase (v16). Alignments were categorized as either exact or loose matches. For loose matches, a custom Smith-Waterman local alignment algorithm was used, with a gap limit of -3, a match score of 2, and a mismatch penalty of -1. No more than 3 mismatches were allowed and the cutoff score used for this algorithm was 1.46 [20]. Sequencing reads that did not map to a known miRNA were submitted for analysis with our novel miRNA discovery platform.

### Quantitative real-time PCR

Validation with quantitative real-time PCR (qPCR) was performed using cDNA from non-treated (ethanol control) and CLA t10,c12 treated ovarian cancer cells (A2780). Pre-validated RT^2^ miRNA qPCR assays were purchased from Qiagen: *hsa-miR-184* (catalog number: MPH00070A-200), *hsa-miR-215* (catalog number: MPH00111A-200), *hsa-miR-143* (catalog number: MPH01177A-200), and *RNU6-2* (catalog number: MPH01653A-200). All reactions were done in triplicate with three biological repeats. RUN6-2 was used as endogenous control. qPCR was performed in iCycler-myIQ5 (Bio-Rad-Hercules, CA) using conditions that have been previously described with RT^2^ SYBR Green Fluor qPCR Mastermix (Qiagen) [19]. *RNU6-2* was used as endogenous control. Fold-change in expression compared to control was calculated using delta-delta CT method and mean fold-change is reported. For qPCR of ER stress markers, we followed the same protocol as published in our earlier study [21]. S27 was used as the housekeeping gene for normalization of the ER stress markers.

### Statistical analysis

All experiments were independently verified in triplicate assays. Continuous variables were compared using Student’s *t* test (between two groups) or analysis of variance (for all groups) if normally distributed. In nonparametric values, continuous variables were compared with the use of the Mann-Whitney U test or Kruskal-Wallis test (for all groups). The statistical significance of the data was determined by using the Statistical Package for Social Scientists software (SPSS, Inc., version 17.0, Chicago, IL).

## Results

### Effects of CLA on cell viability and proliferation

**Fig 1:**
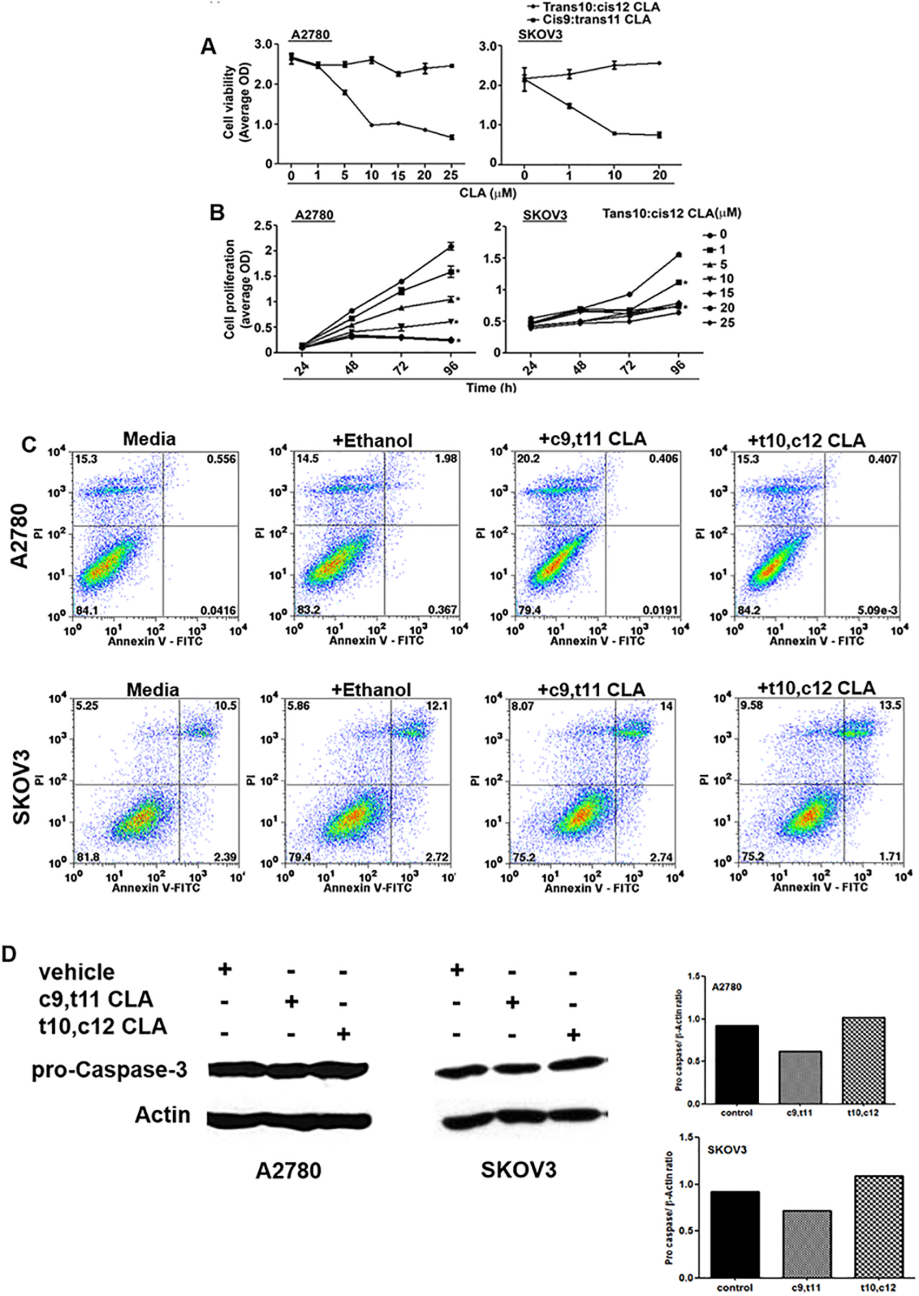
t10,c12 CLA inhibits the proliferation and viability of ovarian cancer cells but does not induce apoptosis. (A) Cell viability was determined in SKOV-3 and A2780 cells treated with increasing concentrations of either c9,t11 CLA or t10,c122. After 72 h incubation with these CLA isomers, the effect on the viability of the cells was measured by conducting the MTT assay. (B) Time course experiment was conducted to determine the effect of t10,c12 CLA on the proliferation of SKOV-3 and A2780 cells. The cells were treated with increasing concentrations of t10,c12 CLA for 24, 48 and 72 h and monitored by MTT assay. A steady increase in the absorbance was observed with increasing time of incubation due to proliferation. Results in *A* and *B* are mean of three independent experiments. In each independent experiment, there were eight replicates of the test and control treatments. (C) A2780 (upper panel) and SKOV3 (lower panel) cells were cultured in media, or media containing ethanol (vehicle control), c9,t11 CLA (7 μM) or t10,c12 CLA (7 μM). After incubation for 72 h, the A2780 and SKOV3 cells were stained with Annexin V and propidium iodide and analyzed for apoptosis by flow cytometry. Dot plots shown are representative data from three independent experiments. (D) The apoptotic status of the t10,c12 CLA treated A2780 and SKOV3 cells was also determined by monitoring the expression of pro-caspase3. The cancer cells were incubated with media containing ethanol (vehicle), c9,t11 CLA (7 μM), or t10,c12 CLA (7 μM) for 72 h. Following incubation, the cells were lysed and pro-caspase3 and actin (loading control) were monitored by western blotting. Western blotting data is representative of three independent experiments. The bar graphs show quantitation of the western blots for both cell lines.

We screened SKOV-3 and A2780 cells for their response to the two CLA isomers at concentrations between 0–50 μM (data not shown). No inhibition of SKOV3 and A2780 viability and proliferation was observed when the cells were treated with c9,t11 CLA (data not shown). On the contrary, t10,c12 CLA inhibited the proliferation of both cell lines at an IC_50_ of 7 μM (Fig 1A). Since, in clinical trials of mixed isomers of CLA, it has been shown that systemic concentrations as high as 43 μM can be achieved following daily oral administration of this fatty acid [22, 23], we concluded that the IC_50_ concentration at which t10,c12 CLA was inhibiting the viability and proliferation of the ovarian cancer cells was pharmacologically attainable in cancer patients. However, t10,c12 CLA was not inducing apoptosis in A2780 and SKOV-3 cells as determined by Annexin V assays and western blot monitoring of cleaved caspase 3 (Fig 1B–1E).

### Gene expression analysis of t10,c12 CLA treated cells

**Fig 2:**
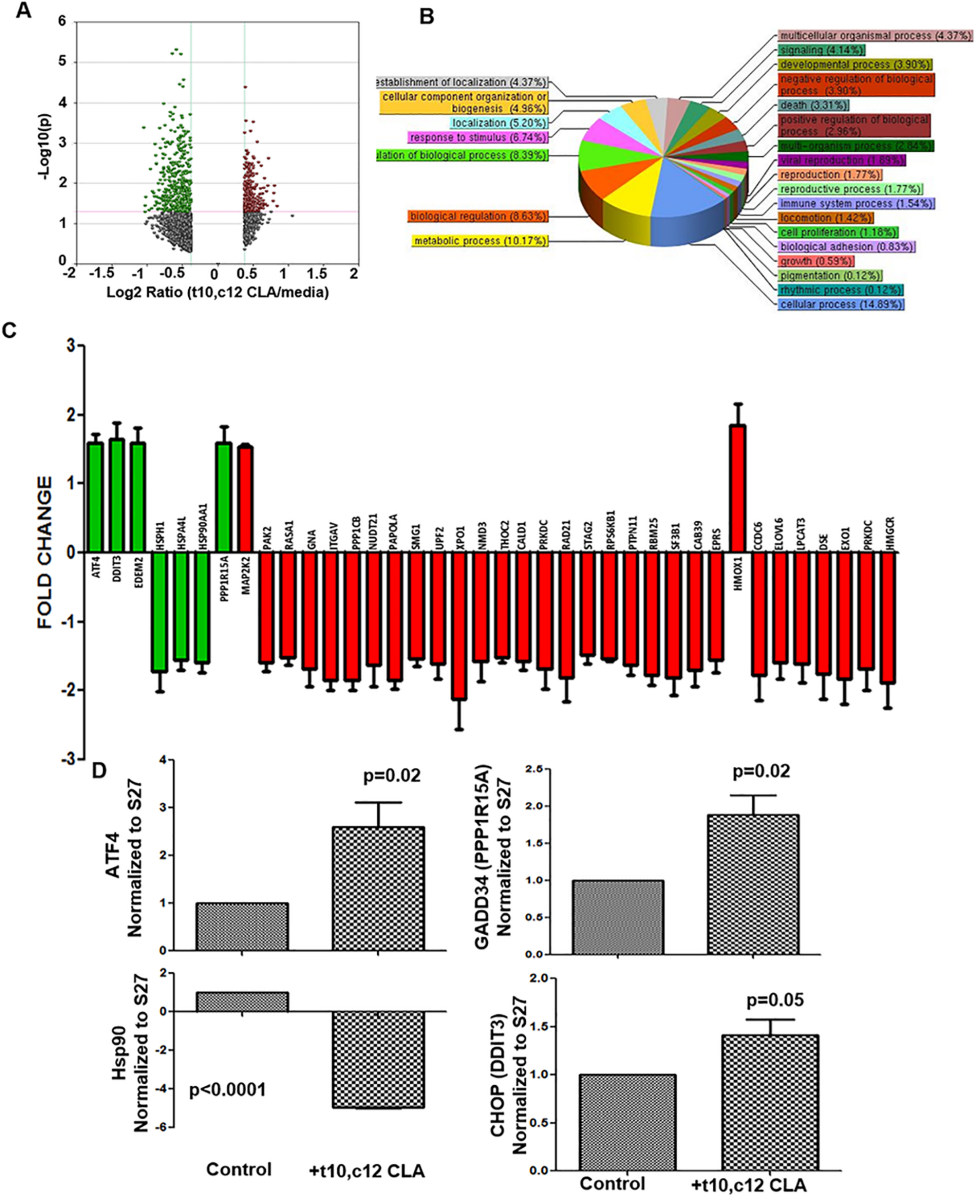
Effect of t10,c12 CLA on the global gene expression shows significant changes in critical genes from the ER stress pathway. A2780 cells were treated with 7 μM t10,c12 CLA for 72 h. Control cells were treated with vehicle under identical conditions. RNA was isolated from the t10,c12 CLA-treated cells and the vehicle controls. After quality check of the RNA, the gene expression was determined using the Illumina HT12V4 expression bead chip array. (A) A volcano plot of the differentially expressed genes is shown. Genes that were either up- or down-regulated at or above a 1.5-fold threshold were considered for further analysis. Results shown are mean of three independent experiments. (B) The Gene Ontology (GO) analysis was conducted on the genes that were differentially expressed by at least 1.5-fold (with p<0.02) in t10,c12 CLA-treated A2780 cells. The analysis shows that genes in several critical processes are affected. The numbers in parenthesis denote the percentage of the total differentially expressed genes that belong to the individual cellular processes. (C) The fold-change in expression of the most prominent genes detected in the t10,c12 CLA-treated A2780 cells is shown. Significantly, based on the fold change, top 7 differentially expressed genes (ATF4, CHOP (DDIT3), EDEM2, HSPH1, HSPA4L, HSP90AA1, PPP1R15A, and MAP2K2) shown in this bar plot belong to the ER stress pathway (green bars). (D) Independent validation of the differential expression of ER stress-related genes, ATF4, GADD34, Hsp90 and CHOP (DDIT3) in t10,c12 CLA treated ovarian cancer cells was monitored by real-time quantitative PCR. The A2780 cells were treated with t10,c12 CLA (7 μM) for 72 h. Mean readings from three separate experiments is plotted. Expression of the genes is normalized to the housekeeping gene, S27.

**Fig 3:**
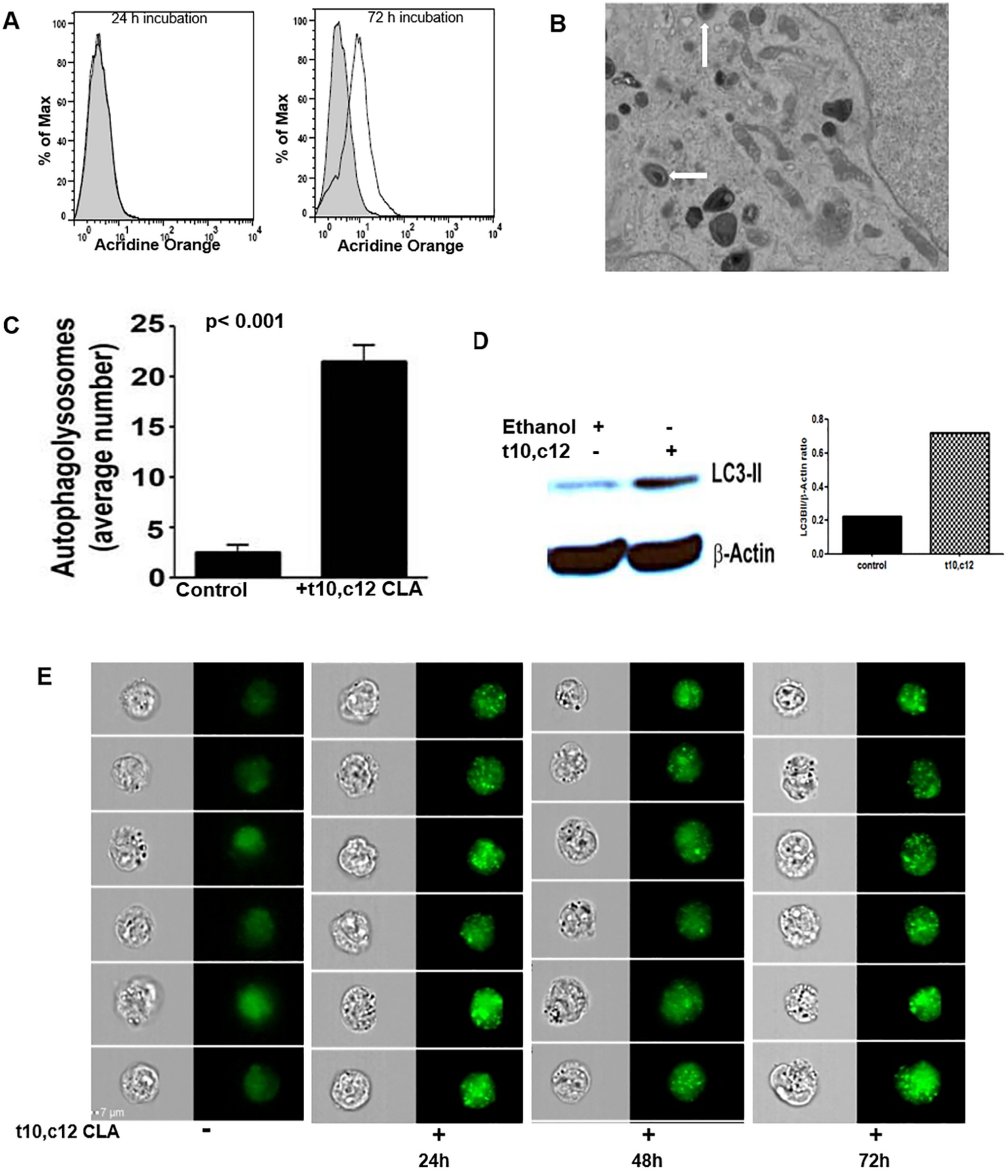
t10,c12 CLA induces autophagy in ovarian cancer cells. A2780 cells were treated with vehicle (control) or t10,c12 CLA (7 μM) for 24 or 72 h. (A) Following incubation with the CLA isomer (Solid line) or the vehicle control (shaded histogram), the cells were harvested and stained with acridine orange. The extent of acridine orange staining in the cells was measured by flow cytometry. Histograms are representative of three independent experiments. (B) Autophagosomes (denoted by white arrows) were detected by electron microscopy in t10,c12 CLA treated cells. Representative image is shown. (C) Counting of the microscopy images provided quantitative analysis of increased autophagosome formation in the t10,c12 CLA treated cells. Mean of three independent measurements is shown. (D) The microscopy data was confirmed by detecting LC3-II levels by western blotting in control (lane 1) and t10,c12 CLA (lane 2) treated A2780 cells and the band intensity of LC3II normalized to housekeeping protein Actin are shown in the bar graph. Blots shown are representative of three independent Western blotting experiments. (E) Untreated and t10,c12 CLA treated A2780 cells were harvested and stained intracellularly for LC3II. After staining cells were analyzed using Imagestream, the imaging flow cytometer to visualize the LC3II puncta (green). Time point data shows that number of Puncta for LC3II increases with time of treatment. Corresponding bright field and fluorescent images of the treated and untreated cells are shown. Data shown is representative of three independent imaging cytometry experiments.

To identify the mechanism through which t10,c12 CLA was inhibiting proliferation, we determined the effect of this lipid on gene expression in the cancer cells. RNA from A2780 cells treated for 72 h with t10,c12 CLA (7 μM) was isolated and microarray analysis was conducted. A2780 cells maintained in cell culture media devoid of CLA isomer were used as controls. Analysis of microarray data led to identification of 193 genes that were differentially expressed in the t10,c12 CLA treated A2780 cells (Fig 2A and S1 Table). The majority (165) of these differentially expressed genes were downregulated. The prominent pathways altered in ovarian cancer cells as a result of t10,c12 CLA treatment are shown in Fig 2B and S2 Table. ER stress related genes, ATF4, CHOP, GADD34, Hsp90, and EDEM2 were among the top dysregulated genes in the t10,c12 CLA-treated A2780 cells (Fig 2C and S1 Table) [24–27]. Real time quantitative PCR confirmed that ATF4 was upregulated and Hsp90 was downregulated in the t10,c12 CLA-treated cells. GADD34 and CHOP are important regulators of ER stress. In the microarray analysis, both GADD34 and CHOP did not show >1.5-fold increase in expression and were therefore not included in Fig 2C. However, we were able to see an increase in both these ER stress markers by quantitative PCR in the t10,c12 CLA-treated cells (Fig 2D). *t10*,*c12 CLA induces autophagy*. Since the ovarian cancer cells were not undergoing apoptosis, we tested if t10,c12 CLA was inducing autophagy in the ovarian cancer cells. As an initial screen, we monitored acridine orange staining in A2780 cells that were treated with t10,c12 CLA (7 μM). After 24 h treatment with t10,c12 CLA no increase in acridine orange staining was observed (Fig 3A). However, after incubation of A2780 cells with t10,c12 CLA for 72 h a 50% increase in acridine orange staining was detected in comparison to controls (Fig 3A), suggesting an increase in autophagosomes.

Next, A2780 cells were treated with t10,c12 CLA (7 μM) for 72 h (time point determined based on the acridine orange assay, Fig 3A) and the cells were monitored for autophagosome formation by electron microscopy. On average, approximately 20 autophagosomes (identified by presence of characteristic double walled membrane) were detected per field when t10,c12 CLA-treated A2780 cells were analyzed by electron microscopy (Fig 3B and 3C). In contrast, <5 autophagosomes were detected per field in control A2780 cells (Fig 3C). These data indicated an increase in autophagy in t10,c12 CLA treated cancer cells. Additional proof that t10,c12 CLA was inducing autophagy was obtained from Western blot showing an approximately 50% increase in the expression level of LC3-II (Fig 3D). LC3 is a 17Kd soluble protein distributed ubiquitously in cells. During autophagy, when cytosolic proteins and organelles are engulfed by autophagosomes, a cytosolic form of LC3 (LC3-I) is conjugated to phosphatidylethanolamine to form LC3-phosphatidylethanolamine conjugate (LC3-II), which is recruited to autophagosomal membranes. When autophagosome fuse with lysosome, intra-autophagosomal components including LC3II are degraded by lysosomal hydrolases. Thus, lysosomal turnover of the autophagosomal marker LC3-II reflects autophagic activity and autophagic death. Fluorescence imaging of autophagic cells shows puncta of LC3-II. LC3-II puncta were detected in t10,c12 CLA treated cells at all three time points (24, 48 and 72 h) tested (Fig 3E). In contrast, there was no LC3-II puncta formation in control cells cultured for 72 h in media alone.

### t10,c12 CLA treatment arrests ovarian cancer cells in G1 phase of the cell cycle

**Fig 4:**
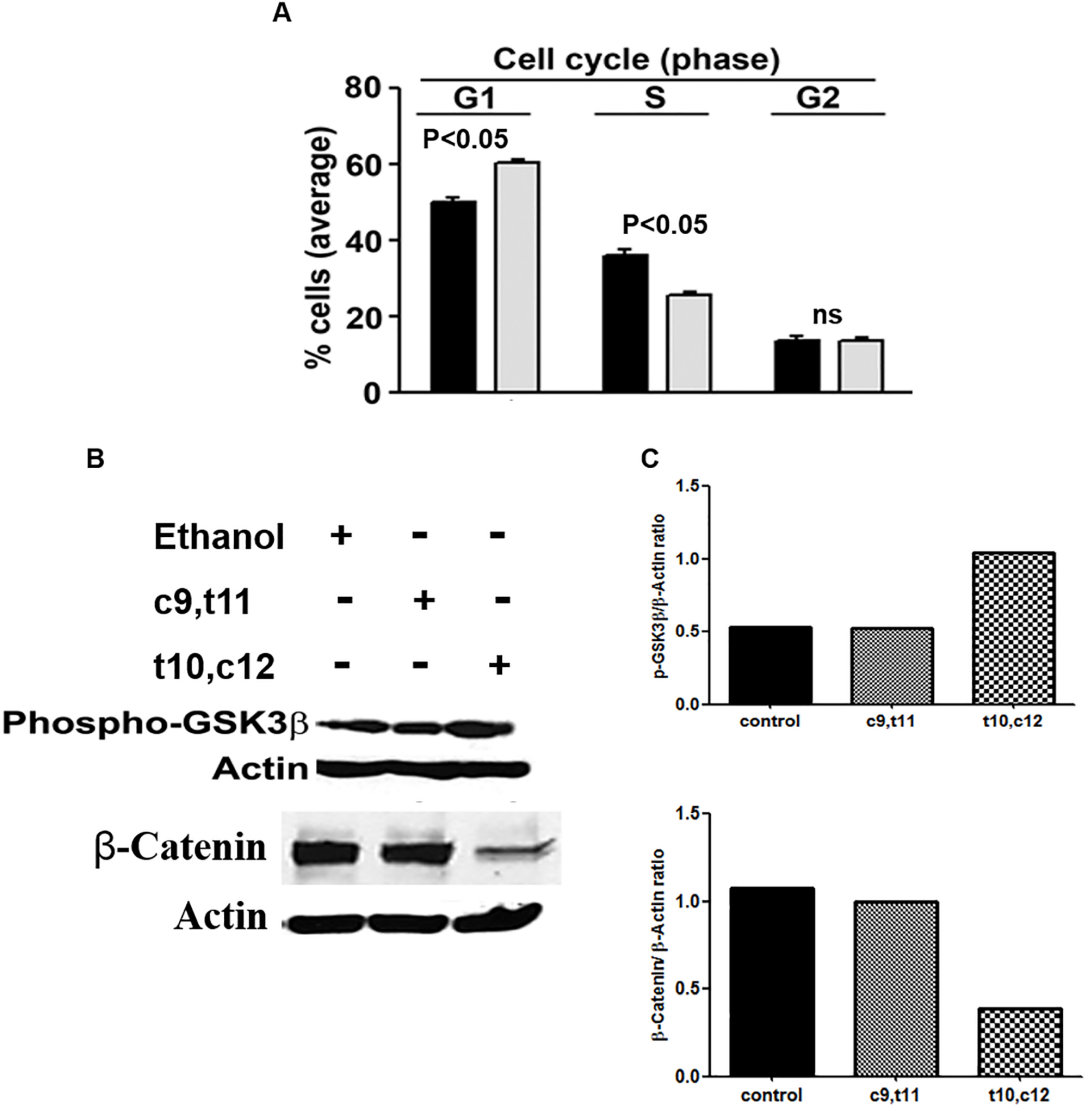
t10,c12 CLA induces G1 cell cycle arrest in ovarian cancer cells. (A) Cell cycle status of vehicle (solid bars) or t10,c12 CLA (7 μM, 72 h; grey bars) treated A2780 cells is shown. *p<0.05 compared to corresponding control. Average data from three independent experiments is shown in the figure. (B) t10,c12 CLA activates GSK3β and induces β-catenin degradation. A2780 cells were incubated with vehicle (lane 1, c9,t11 CLA (lane 2), or t10,c12 CLA (lane 3) for 15 min. Cell lysates were analyzed for GSK3β phosphorylation and β-catenin expression. (C) The bar graph is showing the expression of pGSK3β and β-catenin normalized to the expression of actin in each sample. Western blotting data is representative of three independent observations.

To further explore factors contributing to the inhibition of viability in t10,c12 CLA treated ovarian cancer cells, cell cycle analysis was conducted. A2780 cells treated with t10,c12 CLA for 72 h and stained with propidium iodide showed an approximately 15–20% increase in the cells in the G1 phase and a corresponding 10–15% decrease in the S phase of the of the cell cycle (Fig 4A).

### t10,c12 CLA inhibits β-catenin expression by modulating GSK3β phosphorylation

ER stress is responsible for activating the ubiquitously expressed glycogen synthase kinase 3-β (GSK3β). Western blotting of lysates of A2780 cells treated with t10,c12 CLA showed that there was ~50% increase in the phosphorylation of this enzyme as compared to the untreated control cells (Fig 4B and 4C).

Activation of GSK3β leads to phosphorylation of β-catenin, an important transcription factor regulating the G1/S transition by cancer cells. The phosphorylated β-catenin is tagged for proteosomal degradation whereby it is unable to mediate the transcription of responsive genes [28]. Indeed, t10,c12 CLA treatment resulted in a >70% loss in the signal for β-catenin as compared to the untreated control and c9,t11 CLA-treated A2780 cells (Fig 4B and 4C).

### t10,c12 CLA upregulates expression of miR184 and miR215

**Fig 5:**
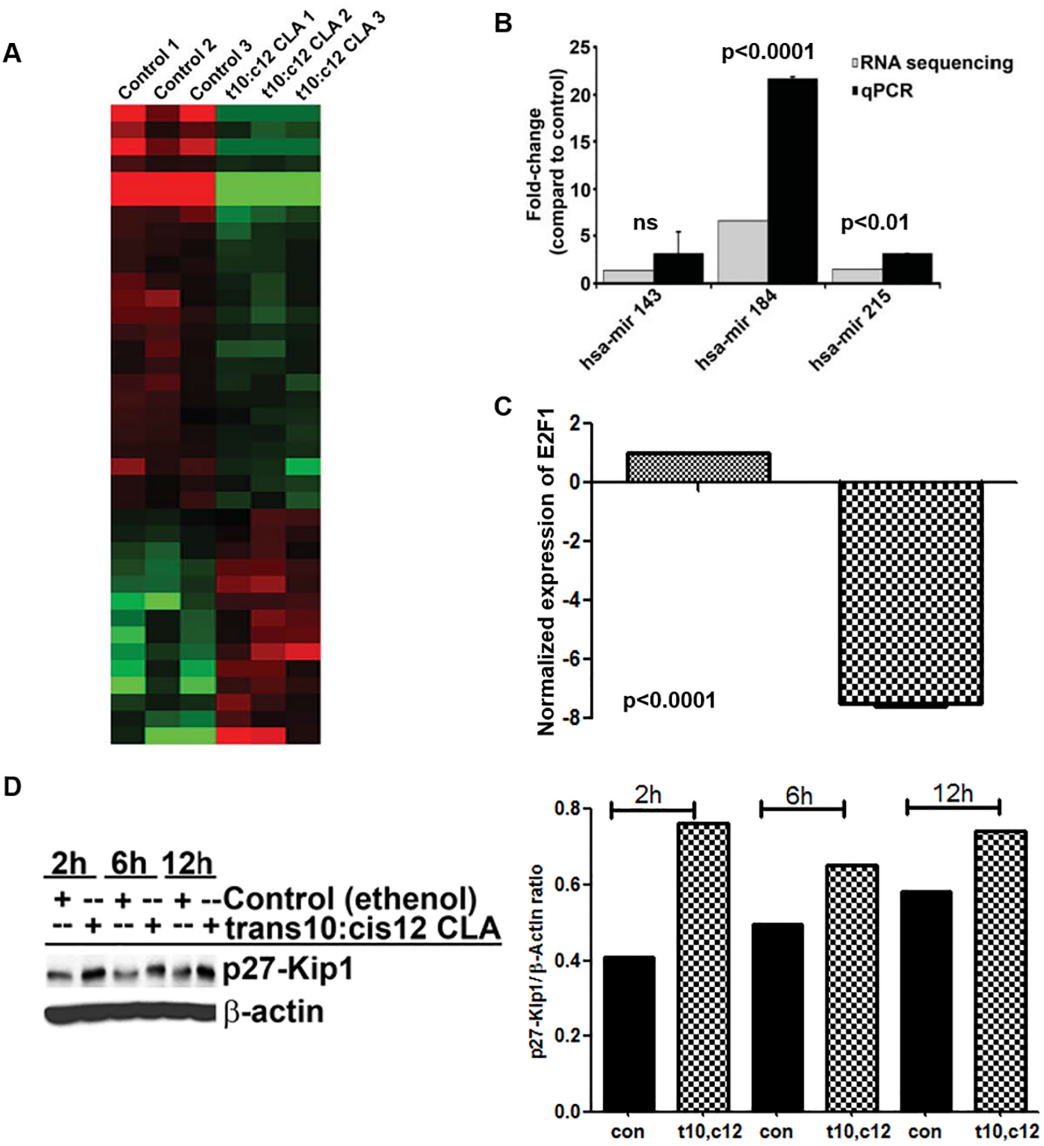
Effect of t10,c12 CLA on microRNA expression. (A) Total RNA was prepared from three control and three t10,c12 CLA (7 μM) treated cultures of A2780 cells and used to perform small RNA sequencing. After normalization, read counts for known human miRNAs were compared. Mature miRNA transcripts with > 3-fold change (*P*<0.01) are depicted in the heat map. The 46 miRNAs that were differentially expressed are described in S1 Fig. (B), shows fold-increase in miR-184 and miR-215 in response to t10,c12 CLA as analyzed by sequencing microarray and qPCR. (C and D) A2780 cells were treated with t10,c12 CLA (7 μM) for 6 h or for the designated time points. The expression of E2F1 was monitored by real-time qPCR (C) and p27/Kip1 was followed by western blotting (D). (E) Intensity of the p27/KIP1 normalized with actin is shown is a graphical representation of the western blots shown in (D). Western blot are representative of three independent experiments.

Further studies led us to define additional mechanisms that link t10,c12 CLA to cell cycle arrest. These additional mechanisms were first identified through global sequencing of the microRNAs isolated from t10,c12 CLA (7 μM for 24 h) treated A2780 cells and matching untreated controls. A total of 2,038 microRNA were sequenced (Fig 5A). t10,c12 CLA treatment resulted in >1.5-fold up- or down regulation of 46 microRNAs (p<0.001) compared to control (S1 Fig). Two of the miRNAs, miR184 and miR215, were upregulated 6.6 and 1.5-fold, respectively, in the t10,c12 CLA treated cells as compared to controls (Fig 5B). The upregulation of these two miRNAs was validated by real time PCR (Fig 5B). These two miRNAs were specifically selected because of previous reports linking their upregulation to cell cycle arrest.

### miR184 upregulation is associated with E2F1 downregulation in t10,c12 CLA-treated cells

One of the targets of miR184 is the transcription factor E2F1. E2F1 complexes with retinoblastoma gene (Rb) and promotes cell proliferation [29]. Real time PCR experiments proved that the expression of E2F1 was significantly inhibited in A2780 cells that were treated with t10,c12 CLA (Fig 5C).

### miR215 overexpression in t10,c12 CLA treated cells is associated with increased expression of p27/Kip-1

miR215 when complexed with miR192 results in the upregulation of the cyclin-dependent kinase inhibitor, p27-Kip1 [30]. In the microRNA sequencing experiments we observed a marginal (1.2-fold) increase in the expression of miR192. Corresponding with the increase in the expression of miR215 and miR192, we also observed that t10,c12 CLA treatment resulted in an increase in the expression of p27/Kip-1, as detected by Western blotting (Fig 5D and 5E).

### t10,c12 CLA inhibits the invasion and migration of ovarian cancer cells by inhibiting the activation of Src

**Fig 6:**
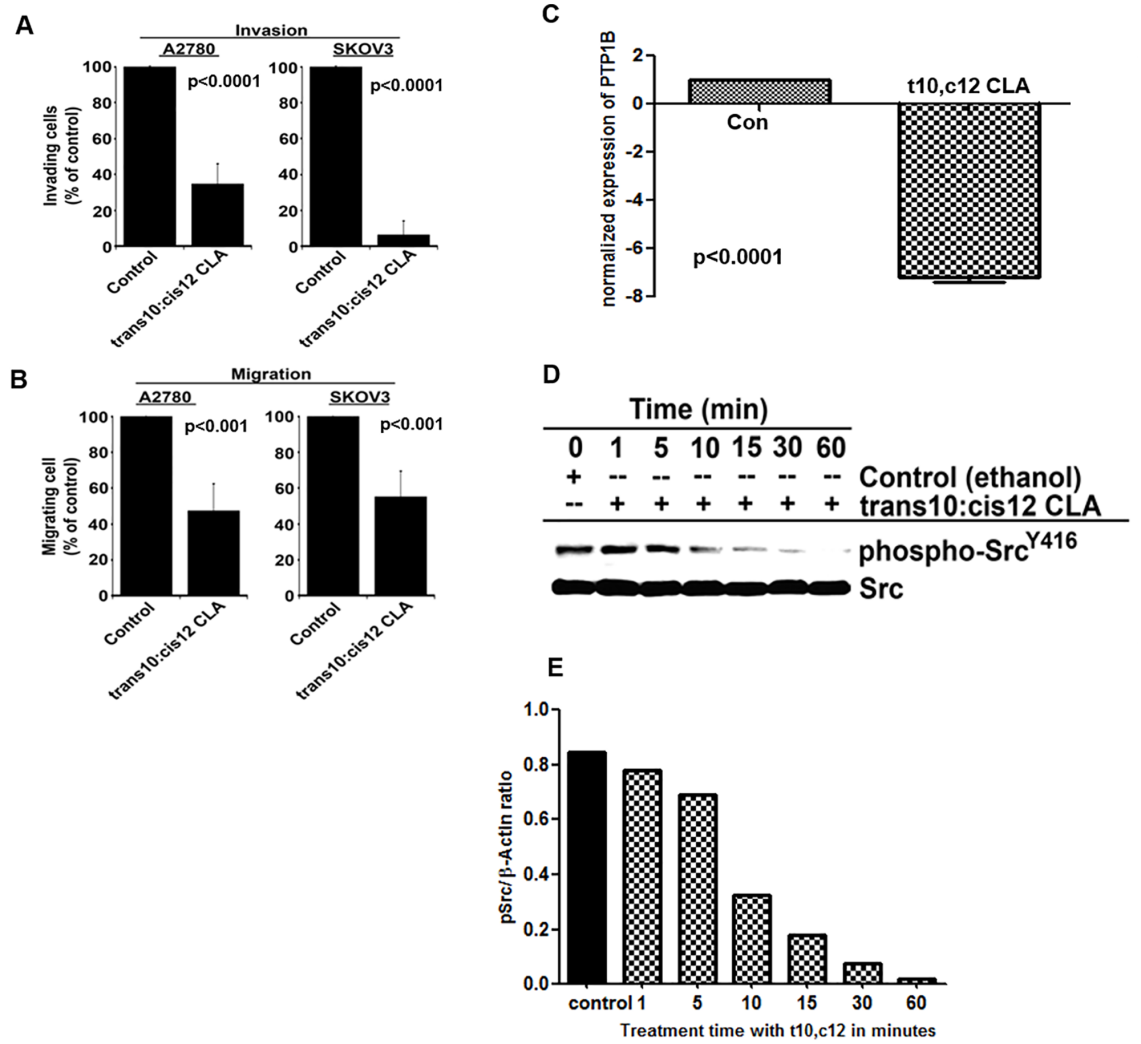
t10,c12 CLA inhibits invasion and migration of ovarian cancer cells. A2780 cells exposed to t10,c12 CLA (7 μM) or to vehicle control for 72 h were monitored for invasion and migration. The cells were plated in a transwell chamber and their ability to (A) invade into a defined extracellular matrix or (B) migrate in response to a chemoattractant was determined. The number of cells invading or migrating through the transwell chamber were counted. Ovarian cancer cells that migrated or invaded were quantified. Each bar is a mean of three separate experiments. P>0.01. (C) mRNA from the A2780 cells was isolated after 3h of t10,c12 CLA (7 μM) treatment, following which, real-time qPCR was used to monitor the expression levels of PTP1B. (D) A2780 cells were treated with the t10,c12 CLA for the designated time points. The cells were lysed and the lysate were monitored for total Src and Tyr 416 phospho-Src. (E) The graphical representation of the western blot data (D) is shown in the bar graph where the band intensity of p-Src is normalized to the Actin. Western blot results are representative of three independent experiments.

t10,c12 CLA also inhibited the invasion and migration of SKOV-3 and A2780 cells by 70–90% and 50–60%, respectively (Fig 6A and 6B). Src kinase plays an important role in modulating proliferation and invasion and migration of cancer cells. In our microarray experiments we had observed a decrease in the expression of the protein tyrosine phosphatase PTP1B. Decrease in the expression of PTP1B was validated by real time PCR (Fig 6C). This enzyme removes the inhibitory phosphate group from Tyr^530^ of Src [31]. Decreased expression of PTP1b therefore likely interferes with the activation of Src. t10,c12 CLA also has an immediate effect on Src function. A rapid decrease in phosphorylaton of Tyr^416^ of Src was observed as early as 10 min after the A2780 cells were treated with t10,c12 CLA (7 μM) (Fig 6D and 6E).

## Discussion

The current study demonstrates that t10,c12 CLA is an inhibitor of the viability, invasion and migration of ovarian cancer cells. t10,c12 CLA inhibited the viability of SKOV-3 and A2780 cells with an IC_50_ of approximately 7 μM. Data from human clinical trials where preparations containing mixed isomers of CLA were administered, serum concentration of this fatty acid were determined to be as high as 50 μM [22]. Therefore, the inhibition of viability of ovarian cancer is achievable at pharmacologically relevant concentration of this fatty acid.

Studies conducted in breast and other cancer models have indicated that t10,c12 CLA caused cell death in the cancer cells due to apoptosis. In contrast to previous reports, we did not observe apoptosis in t10,c12 CLA-treated cells (Fig 1C). Instead, decreased proliferation observed in our experiments is a direct result of autophagy and cell cycle arrest in cancer cells treated with t10,c12 CLA (Figs 3 and 4A). These results suggest that the ultimate biologic outcome of t10,c12 CLA and the molecular pathways that lead to this result are likely to depend on the type and genetic make-up of cancer cells. Additional studies will be required to understand why even after 72 h of treatment of ovarian cancer cells with t10,c12 CLA, the cells continue to be autophagic and do not undergo cell death.

Another important issue that needs to be addressed is the potential tumor promoting effect of t10,c12 CLA in murine mammary cancer [32]. Erb2 overexpressing mice receiving t10,c12 CLA in their feeds developed mammary hyperplasia and there was accelerated development of breast tumors. Overexpression of Erb2 and the fact that the mice were chronically fed t10,c12 CLA right after weaning are important aspects of this study that may contribute to the pro-tumor effects observed with the CLA isomer. It needs to be examined if long term administration of t10,c12 CLA would lead to enhanced Erb2 signaling and promote ovarian cancer.

CHOP and GADD34 are upregulated in the t10,c12 CLA treated cells (Fig 2D). GADD34 dephosphorylates eIF2α [27]. Perturbation of ER homeostasis leads to increase in misfolded proteins. As a result, the misfolded proteins bind to the chaperone protein BiP causing this chaperone to dissociate from its complex with PERK, ATF6, and IRE1 [24, 33, 34]. Dissociation of BiP activates PERK, ATF6, and IRE1. Activated PERK phosphorylates and thereby activates translation initiation factor eIF2α [25]. Once activated, eIF2α inhibits transcription of majority of the genes. In this context it is worth noting that 165 genes were downregulated as compared to only 28 that were upregulated in the t10,c12 CLA treated ovarian cancer cells. Prominent among these upregulated proteins are ATF4, GADD34, and CHOP.

**Fig 7:**
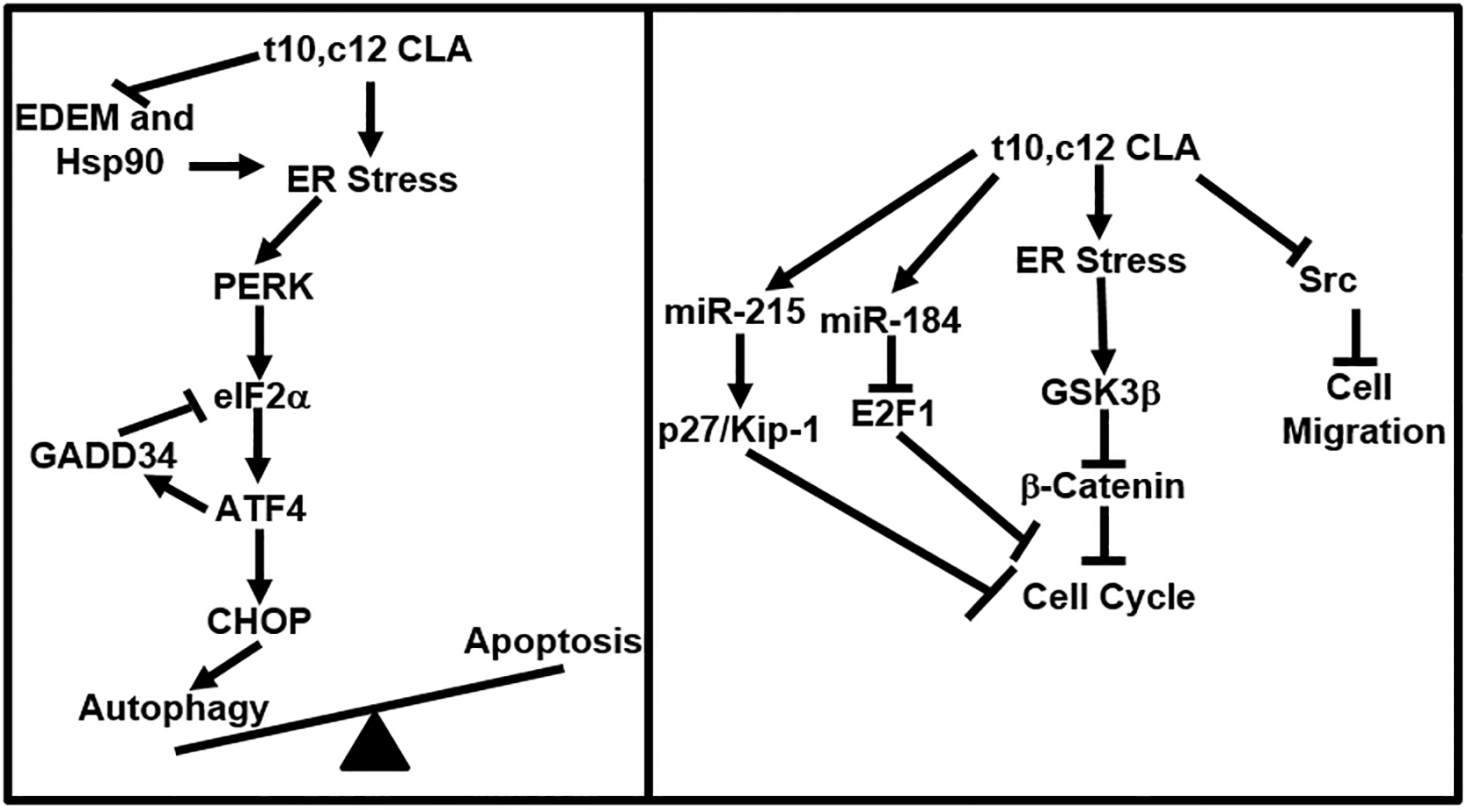
Model describing the mechanism of action of t10,c12 CLA. Induction of ER stress causes activation of the PERK-eIF2α pathway and leads to autophagy. Induction of miR184 and miR215 inhibits cell cycle whereas inhibition of Src causes a decrease in cell migration of t10,c12 CLA-treated cancer cells.

To relieve ER stress, elimination of misfolded proteins through ER Associated protein Degradation (ERAD) is an essential step [35]. EDEM2 and Hsp90 are required for a successful ERAD response [36, 37]. t10,c12 CLA decreased the expression of Hsp90 (Fig 2D) and EDEM2 (S1 Table), suggesting that t10,c12 CLA interferes with ERAD and thereby locks the cancer cells in a persistent state of ER stress (Fig 7).. Even as GADD34 upregulation is expected to provide a negative feedback by dephosphorylating eIF2α, the continued presence of misfolded proteins in the ER may cause chronic activation of the PERK-eIF2α axis. Our on-going studies are focused on understanding the capacity of the t10,c12 CLA treated ovarian cancer cells to endure persistent ER stress.

We have identified two independent mechanisms that contribute to G1 cell cycle arrest in t10,c12 CLA-treated cells- loss of β-catenin and activation of GSK3β (Fig 4B). Additionally, increased expression of miR184 and miR215 also contributes to cell cycle arrest (Fig 5B). Increase in expression of miR184 correlates to downregulation of its target E2F1 and inhibition of miR215 is associated with increases in p27-Kip-1 expression. E2F1 is a transcription factor that when complexed with retinoblastoma protein Rb is unable to initiate transcription resulting in G1 arrest in the cell [38, 39]. t10,c12 CLA by inhibiting the expression of E2F1 is causing cell cycle arrest in the ovarian cancer cells. Furthermore, miR215 and miR192 together can also induce cell cycle arrest and increase expression of p27-Kip1 [30]. Western blot analysis has shown that t10,c12 CLA increases the expression of p27/Kip1 in the ovarian cancer cells. At this point it is not clear if the increase in miR184 and miR215 are a result of ER stress or due to other molecular events mediated by t10,c12 CLA.

Finally, we have also demonstrated that t10,c12 CLA inhibits migration of ovarian cancer cells (Fig 6A and 6B). This observation is congruent with previous studies where administration of CLA was shown to attenuate metastasis of different types of cancer cells in *in vivo* mouse models [40, 41]. Phosphorylation of Tyr^416^ accompanied by dephosphorylation at Tyr^530^ is required for activation of Src [31]. While we observed that Tyr^416^ is phosphorylated to a significantly greater extent in t10,c12 CLA treated cells, the expression of PTP1B, the phosphatase that dephosphorylates Tyr^530^ of Src, is inhibited by t10,c12 CLA.

Overall, the data presented here show that t10,c12 CLA has a multifaceted response in ovarian cancer cells. We propose that t10,c12 CLA should be especially considered for chronic administration in patients in remission for this disease after their initial cytoreductive surgeries and chemotherapy. Such a maintenance therapy regimen will result in inhibition of the growth and peritoneal spread of the cancer and extend the period of cancer-free survival in ovarian cancer patients.

## Supporting information

S1 Table Microarray data analysis showing differentially expressed genes in control versus t10,c12 CLA treated A2780 cells. (DOCX)

S2 Table This table shows changes in gene expression in prominent pathways when A2780 cells were treated with t10,c12 CLA. Z-score is the measure of standard deviation. A Z-score of 2 indicates that the value is two standard deviations away from mean.
(DOCX)

S1 Fig Fold change in expression of the 46 differentially expressed miRNA in t10,c12 CLA treated A2780 cells as compared to untreated controls is shown in the bar charts. (PDF)

## References

[1] Pariza MW, Ha YL. Conjugated dienoic derivatives of linoleic acid: a new class of anticarcinogens. Med Oncol Tumor Pharmacother. 1990;7(2–3):169–71. . 2232933 10.1007/BF02988544

[2] Ha YL, Grimm NK, Pariza MW. Anticarcinogens from fried ground beef: heat-altered derivatives of linoleic acid. Carcinogenesis. 1987;8(12):1881–7. . 3119246 10.1093/carcin/8.12.1881

[3] Kelley NS, Hubbard NE, Erickson KL. Conjugated linoleic acid isomers and cancer. J Nutr. 2007;137(12):2599–607. . 18029471 10.1093/jn/137.12.2599

[4] Beppu F, Hosokawa M, Tanaka L, Kohno H, Tanaka T, Miyashita K. Potent inhibitory effect of trans9, trans11 isomer of conjugated linoleic acid on the growth of human colon cancer cells. J Nutr Biochem. 2006;17(12):830–6. doi: 10.1016/j.jnutbio.2006.01.007 . 16563722 10.1016/j.jnutbio.2006.01.007

[5] Ip C, Dong Y, Ip MM, Banni S, Carta G, Angioni E, et al Conjugated linoleic acid isomers and mammary cancer prevention. Nutr Cancer. 2002;43(1):52–8. doi: 10.1207/S15327914NC431_6 . 12467135 10.1207/S15327914NC431_6

[6] Yamasaki M, Chujo H, Koga Y, Oishi A, Rikimaru T, Shimada M, et al Potent cytotoxic effect of the trans10, cis12 isomer of conjugated linoleic acid on rat hepatoma dRLh-84 cells. Cancer Lett. 2002;188(1–2):171–80. . 12406562 10.1016/s0304-3835(02)00459-7

[7] Ou L, Wu Y, Ip C, Meng X, Hsu YC, Ip MM. Apoptosis induced by t10,c12-conjugated linoleic acid is mediated by an atypical endoplasmic reticulum stress response. J Lipid Res. 2008;49(5):985–94. doi: 10.1194/jlr.M700465-JLR200 . 18263853 10.1194/jlr.M700465-JLR200 PMC2311448

[8] Cho HJ, Kim EJ, Lim SS, Kim MK, Sung MK, Kim JS, et al Trans-10,cis-12, not cis-9,trans-11, conjugated linoleic acid inhibits G1-S progression in HT-29 human colon cancer cells. J Nutr. 2006;136(4):893–8. . 16549447 10.1093/jn/136.4.893

[9] Miller A, Stanton C, Devery R. Modulation of arachidonic acid distribution by conjugated linoleic acid isomers and linoleic acid in MCF-7 and SW480 cancer cells. Lipids. 2001;36(10):1161–8. . 11768161 10.1007/s11745-001-0827-0

[10] Flowers M, Thompson PA. t10c12 conjugated linoleic acid suppresses HER2 protein and enhances apoptosis in SKBr3 breast cancer cells: possible role of COX2. PLoS One. 2009;4(4):e5342 doi: 10.1371/journal.pone.0005342 . 19399184 10.1371/journal.pone.0005342 PMC2671134

[11] Degner SC, Kemp MQ, Bowden GT, Romagnolo DF. Conjugated linoleic acid attenuates cyclooxygenase-2 transcriptional activity via an anti-AP-1 mechanism in MCF-7 breast cancer cells. J Nutr. 2006;136(2):421–7. . 16424122 10.1093/jn/136.2.421

[12] Lim DY, Tyner AL, Park JB, Lee JY, Choi YH, Park JH. Inhibition of colon cancer cell proliferation by the dietary compound conjugated linoleic acid is mediated by the CDK inhibitor p21CIP1/WAF1. J Cell Physiol. 2005;205(1):107–13. doi: 10.1002/jcp.20380 . 15880444 10.1002/jcp.20380

[13] Wang LS, Huang YW, Liu S, Chang HL, Ye W, Shu S, et al Conjugated linoleic acid (CLA) modulates prostaglandin E2 (PGE2) signaling in canine mammary cells. Anticancer Res. 2006;26(2A):889–98. .16619484

[14] Hsu YC, Ip MM. Conjugated linoleic acid-induced apoptosis in mouse mammary tumor cells is mediated by both G protein coupled receptor-dependent activation of the AMP-activated protein kinase pathway and by oxidative stress. Cell Signal. 2011;23(12):2013–20. doi: 10.1016/j.cellsig.2011.07.015 . 21821121 10.1016/j.cellsig.2011.07.015 PMC3265966

[15] Gubbels JA, Claussen N, Kapur AK, Connor JP, Patankar MS. The detection, treatment, and biology of epithelial ovarian cancer. J Ovarian Res. 2010;3:8 doi: 10.1186/1757-2215-3-8 . 20350313 10.1186/1757-2215-3-8 PMC2856581

[16] Shahzad MM, Mangala LS, Han HD, Lu C, Bottsford-Miller J, Nishimura M, et al Targeted delivery of small interfering RNA using reconstituted high-density lipoprotein nanoparticles. Neoplasia. 2011;13(4):309–19. . 21472135 10.1593/neo.101372 PMC3071079

[17] Jones LJ, Gray M, Yue ST, Haugland RP, Singer VL. Sensitive determination of cell number using the CyQUANT cell proliferation assay. J Immunol Methods. 2001;254(1–2):85–98. . 11406155 10.1016/s0022-1759(01)00404-5

[18] Gildea JJ, Harding MA, Gulding KM, Theodorescu D. Transmembrane motility assay of transiently transfected cells by fluorescent cell counting and luciferase measurement. BioTechniques. 2000;29(1):81–6. . 10907081 10.2144/00291st02

[19] Halder J, Landen CN Jr., Lutgendorf SK, Li Y, Jennings NB, Fan D, et al Focal adhesion kinase silencing augments docetaxel-mediated apoptosis in ovarian cancer cells. Clin Cancer Res. 2005;11(24 Pt 1):8829–36. doi: 10.1158/1078-0432.CCR-05-1728 . 16361572 10.1158/1078-0432.CCR-05-1728 PMC3144933

[20] Creighton CJ, Reid JG, Gunaratne PH. Expression profiling of microRNAs by deep sequencing. Briefings in bioinformatics. 2009;10(5):490–7. doi: 10.1093/bib/bbp019 . 19332473 10.1093/bib/bbp019 PMC2733187

[21] Liu Y, Whelan RJ, Pattnaik BR, Ludwig K, Subudhi E, Rowland H, et al Terpenoids from Zingiber officinale (Ginger) induce apoptosis in endometrial cancer cells through the activation of p53. PLoS One. 2012;7(12):e53178 doi: 10.1371/journal.pone.0053178 . 23300887 10.1371/journal.pone.0053178 PMC3534047

[22] Lau DS, Archer MC. The 10t,12c isomer of conjugated linoleic acid inhibits fatty acid synthase expression and enzyme activity in human breast, colon, and prostate cancer cells. Nutr Cancer. 2010;62(1):116–21. doi: 10.1080/01635580903191536 . 20043266 10.1080/01635580903191536

[23] Mosley EE, McGuire MK, Williams JE, McGuire MA. Cis-9, trans-11 conjugated linoleic acid is synthesized from vaccenic acid in lactating women. J Nutr. 2006;136(9):2297–301. . 16920844 10.1093/jn/136.9.2297

[24] Bertolotti A, Zhang Y, Hendershot LM, Harding HP, Ron D. Dynamic interaction of BiP and ER stress transducers in the unfolded-protein response. Nat Cell Biol. 2000;2(6):326–32. doi: 10.1038/35014014 . 10854322 10.1038/35014014

[25] Harding HP, Zhang Y, Bertolotti A, Zeng H, Ron D. Perk is essential for translational regulation and cell survival during the unfolded protein response. Molecular cell. 2000;5(5):897–904. . 10882126 10.1016/s1097-2765(00)80330-5

[26] Harding HP, Zhang Y, Ron D. Protein translation and folding are coupled by an endoplasmic-reticulum-resident kinase. Nature. 1999;397(6716):271–4. doi: 10.1038/16729 . 9930704 10.1038/16729

[27] Novoa I, Zeng H, Harding HP, Ron D. Feedback inhibition of the unfolded protein response by GADD34-mediated dephosphorylation of eIF2alpha. J Cell Biol. 2001;153(5):1011–22. . 11381086 10.1083/jcb.153.5.1011 PMC2174339

[28] Rubinfeld B, Albert I, Porfiri E, Fiol C, Munemitsu S, Polakis P. Binding of GSK3beta to the APC-beta-catenin complex and regulation of complex assembly. Science. 1996;272(5264):1023–6. . 8638126 10.1126/science.272.5264.1023

[29] Gehrke S, Imai Y, Sokol N, Lu B. Pathogenic LRRK2 negatively regulates microRNA-mediated translational repression. Nature. 2010;466(7306):637–41. doi: 10.1038/nature09191 . 20671708 10.1038/nature09191 PMC3049892

[30] Boni V, Bitarte N, Cristobal I, Zarate R, Rodriguez J, Maiello E, et al miR-192/miR-215 influence 5-fluorouracil resistance through cell cycle-mediated mechanisms complementary to its post-transcriptional thymidilate synthase regulation. Mol Cancer Ther. 2010;9(8):2265–75. doi: 10.1158/1535-7163.MCT-10-0061 . 20647341 10.1158/1535-7163.MCT-10-0061

[31] Roskoski R Jr. Src kinase regulation by phosphorylation and dephosphorylation. Biochem Biophys Res Commun. 2005;331(1):1–14. doi: 10.1016/j.bbrc.2005.03.012 . 15845350 10.1016/j.bbrc.2005.03.012

[32] Ip MM, McGee SO, Masso-Welch PA, Ip C, Meng X, Ou L, et al The t10,c12 isomer of conjugated linoleic acid stimulates mammary tumorigenesis in transgenic mice over-expressing erbB2 in the mammary epithelium. Carcinogenesis. 2007;28(6):1269–76. doi: 10.1093/carcin/bgm018 . 17259656 10.1093/carcin/bgm018 PMC2776704

[33] Ni M, Lee AS. ER chaperones in mammalian development and human diseases. FEBS Lett. 2007;581(19):3641–51. doi: 10.1016/j.febslet.2007.04.045 . 17481612 10.1016/j.febslet.2007.04.045 PMC2040386

[34] Shen J, Chen X, Hendershot L, Prywes R. ER stress regulation of ATF6 localization by dissociation of BiP/GRP78 binding and unmasking of Golgi localization signals. Dev Cell. 2002;3(1):99–111. . 12110171 10.1016/s1534-5807(02)00203-4

[35] Maattanen P, Gehring K, Bergeron JJ, Thomas DY. Protein quality control in the ER: the recognition of misfolded proteins. Semin Cell Dev Biol. 2010;21(5):500–11. doi: 10.1016/j.semcdb.2010.03.006 . 20347046 10.1016/j.semcdb.2010.03.006

[36] Oda Y, Hosokawa N, Wada I, Nagata K. EDEM as an acceptor of terminally misfolded glycoproteins released from calnexin. Science. 2003;299(5611):1394–7. doi: 10.1126/science.1079181 . 12610305 10.1126/science.1079181

[37] Marcu MG, Doyle M, Bertolotti A, Ron D, Hendershot L, Neckers L. Heat shock protein 90 modulates the unfolded protein response by stabilizing IRE1alpha. Mol Cell Biol. 2002;22(24):8506–13. doi: 10.1128/MCB.22.24.8506-8513.2002 . 12446770 10.1128/MCB.22.24.8506-8513.2002 PMC139892

[38] Johnson DG, Schwarz JK, Cress WD, Nevins JR. Expression of transcription factor E2F1 induces quiescent cells to enter S phase. Nature. 1993;365(6444):349–52. doi: 10.1038/365349a0 . 8377827 10.1038/365349a0

[39] Qin XQ, Livingston DM, Ewen M, Sellers WR, Arany Z, Kaelin WG Jr. The transcription factor E2F-1 is a downstream target of RB action. Mol Cell Biol. 1995;15(2):742–55. . 7823942 10.1128/mcb.15.2.742 PMC231942

[40] Soel SM, Choi OS, Bang MH, Yoon Park JH, Kim WK. Influence of conjugated linoleic acid isomers on the metastasis of colon cancer cells in vitro and in vivo. J Nutr Biochem. 2007;18(10):650–7. doi: 10.1016/j.jnutbio.2006.10.011 . 17368880 10.1016/j.jnutbio.2006.10.011

[41] Kuniyasu H, Yoshida K, Sasaki T, Sasahira T, Fujii K, Ohmori H. Conjugated linoleic acid inhibits peritoneal metastasis in human gastrointestinal cancer cells. Int J Cancer. 2006;118(3):571–6. doi: 10.1002/ijc.21368 . 16106401 10.1002/ijc.21368

