# Supplementary material for: Trans10,cis12 conjugated linoleic acid inhibits proliferation and migration of ovarian cancer cells by inducing ER stress, autophagy, and modulation of Src
Source: PLoS One. 2018 Jan 11;13(1):e0189524. doi: 10.1371/journal.pone.0189524 (PMC5764254; doi:10.1371/journal.pone.0189524)
Supplement: S1 Table — (DOCX) [file pone.0189524.s001.docx]

**S1 Table**

|  |  |  |  |  |  |  |
| --- | --- | --- | --- | --- | --- | --- |
| **Gene Name** | **Gene ID** | **Ratio** | **Direction** |  |  |  |
| Heme oxygenase (decycling) 1 | HMOX1 | 1.79 | Up |  |  |  |
| PREDICTED: similar to monoglyceride lipase (LOC653521), mRNA. | - | 1.74 | Up |  |  |  |
| CCAAT/enhancer binding protein (C/EBP), beta | CEBPB | 1.73 | Up |  |  |  |
| Testis expressed 19 | TEX19 | 1.73 | Up |  |  |  |
| PREDICTED: hypothetical protein LOC100132394 (LOC100132394), mRNA. | - | 1.64 | Up |  |  |  |
| 28S ribosomal RNA (LOC100008589), non-coding RNA. | - | 1.64 | Up |  |  |  |
| Histone cluster 2, H2aa3 | HIST2H2AA3 | 1.63 | Up |  |  |  |
| DNA-damage-inducible transcript 3 | DDIT3 | 1.61 | Up |  |  |  |
| small nucleolar RNA host gene 7 (non-protein coding) (SNHG7), transcript variant 1, non-coding RNA. | - | 1.59 | Up |  |  |  |
| Activating transcription factor 4 (tax-responsive enhancer element B67) | ATF4 | 1.59 | Up |  |  |  |
| Sequestosome 1 | SQSTM1 | 1.57 | Up |  |  |  |
| Histidine triad nucleotide binding protein 2 | HINT2 | 1.57 | Up |  |  |  |
| ER degradation enhancer, mannosidase alpha-like 2 | EDEM2 | 1.57 | Up |  |  |  |
| Lysophosphatidylcholine acyltransferase 3 | LPCAT3 | 1.57 | Up |  |  |  |
| Chromosome 6 open reading frame 48 | C6orf48 | 1.55 | Up |  |  |  |
| Protein phosphatase 1, regulatory (inhibitor) subunit 15A | PPP1R15A | 1.55 | Up |  |  |  |
| Transmembrane protease, serine 11D | TMPRSS11D | 1.55 | Up |  |  |  |
| Mitogen-activated protein kinase kinase 2 | MAP2K2 | 1.54 | Up |  |  |  |
| small nucleolar RNA host gene 7 (non-protein coding) (SNHG7), transcript variant 2, non-coding RNA. | - | 1.54 | Up |  |  |  |
| PREDICTED: similar to galectin 9 short isoform, transcript variant 2 (LOC654346), mRNA. | - | 1.54 | Up |  |  |  |
| RNA, U1 small nuclear 3 (RNU1-3), small nuclear RNA. | - | 1.53 | Up |  |  |  |
| Interferon stimulated exonuclease gene 20kDa | ISG20 | 1.53 | Up |  |  |  |
| RNA, U1 small nuclear 5 (RNU1-5), small nuclear RNA. | - | 1.52 | Up |  |  |  |
| PREDICTED: hypothetical protein LOC100134364 (LOC100134364), mRNA. | - | 1.52 | Up |  |  |  |
| BRF2, subunit of RNA polymerase III transcription initiation factor, BRF1-like | BRF2 | 1.51 | Up |  |  |  |
| Chromosome 10 open reading frame 35 | C10orf35 | 1.51 | Up |  |  |  |
| major histocompatibility complex, class I, H (pseudogene) | - | 1.5 | Up |  |  |  |
| Discs, large homolog 4 (Drosophila) | DLG4 | 1.5 | Up |  |  |  |
| Lin-28 homolog B (C. elegans) | LIN28B | 2.07 | Down |  |  |  |
| HEAT repeat containing 1 | HEATR1 | 2.04 | Down |  |  |  |
| Solute carrier family 44, member 1 | SLC44A1 | 2.04 | Down |  |  |  |
| Exportin 1 (CRM1 homolog, yeast) | XPO1 | 2.03 | Down |  |  |  |
| Hypothetical LOC144438 | LOC144438 | 1.96 | Down |  |  |  |
| Topoisomerase (DNA) II alpha 170kDa | TOP2A | 1.94 | Down |  |  |  |
| basic leucine zipper and W2 domains 1 like 1 | - | 1.91 | Down |  |  |  |
| PREDICTED: similar to serine/threonine/tyrosine interacting protein, transcript variant 1 (LOC730432), mRNA. | - | 1.9 | Down |  |  |  |
| Proteasome (prosome, macropain) activator subunit 4 | PSME4 | 1.88 | Down |  |  |  |
| **Gene Name** | **Gene ID** | **Ratio** | **Direction** |  |  |  |
| Nuclear casein kinase and cyclin-dependent kinase substrate 1 | NUCKS1 | 1.86 | Down |  |  |  |
| 3-hydroxy-3-methylglutaryl-Coenzyme A reductase | HMGCR | 1.86 | Down |  |  |  |
| ROD1 regulator of differentiation 1 (S. pombe) | ROD1 | 1.85 | Down |  |  |  |
| Integrin, alpha V (vitronectin receptor, alpha polypeptide, antigen CD51) | ITGAV | 1.84 | Down |  |  |  |
| Poly(A) polymerase alpha | PAPOLA | 1.83 | Down |  |  |  |
| SON DNA binding protein | SON | 1.83 | Down |  |  |  |
| CSE1 chromosome segregation 1-like (yeast) | CSE1L | 1.83 | Down |  |  |  |
| LIM domain containing preferred translocation partner in lipoma | LPP | 1.83 | Down |  |  |  |
| BTAF1 RNA polymerase II, B-TFIID transcription factor-associated, 170kDa (Mot1 homolog, S. cerevisiae) | BTAF1 | 1.81 | Down |  |  |  |
| Potassium channel tetramerisation domain containing 3 | KCTD3 | 1.8 | Down |  |  |  |
| Exportin 4 | XPO4 | 1.79 | Down |  |  |  |
| Non-SMC condensin I complex, subunit G | NCAPG | 1.79 | Down |  |  |  |
| Insulin-like growth factor 2 mRNA binding protein 3 | IGF2BP3 | 1.79 | Down |  |  |  |
| Splicing factor 3b, subunit 1, 155kDa | SF3B1 | 1.78 | Down |  |  |  |
| CSE1 chromosome segregation 1-like (yeast) | CSE1L | 1.77 | Down |  |  |  |
| DCP2 decapping enzyme homolog (S. cerevisiae) | DCP2 | 1.77 | Down |  |  |  |
| Protein phosphatase 1, catalytic subunit, beta isoform | PPP1CB | 1.75 | Down |  |  |  |
| RNA binding motif protein 25 | RBM25 | 1.75 | Down |  |  |  |
| Discoidin, CUB and LCCL domain containing 2 | DCBLD2 | 1.75 | Down |  |  |  |
| Nucleosome assembly protein 1-like 1 | NAP1L1 | 1.75 | Down |  |  |  |
| Exonuclease 1 | EXO1 | 1.74 | Down |  |  |  |
| RAD21 homolog (S. pombe) | RAD21 | 1.73 | Down |  |  |  |
| ROD1 regulator of differentiation 1 (S. pombe) | ROD1 | 1.73 | Down |  |  |  |
| Secretory carrier membrane protein 1 | SCAMP1 | 1.72 | Down |  |  |  |
| AT hook containing transcription factor 1 | AHCTF1 | 1.72 | Down |  |  |  |
| ATP-binding cassette, sub-family E (OABP), member 1 | ABCE1 | 1.71 | Down |  |  |  |
| Suppressor of zeste 12 homolog (Drosophila) | SUZ12 | 1.71 | Down |  |  |  |
| DEAD (Asp-Glu-Ala-Asp) box polypeptide 21 | DDX21 | 1.71 | Down |  |  |  |
| Structural maintenance of chromosomes 4 | SMC4 | 1.71 | Down |  |  |  |
| Ubiquitin protein ligase E3A | UBE3A | 1.71 | Down |  |  |  |
| Praja ring finger 2 | PJA2 | 1.71 | Down |  |  |  |
| SIX homeobox 4 | SIX4 | 1.71 | Down |  |  |  |
| ATPase family, AAA domain containing 2 | ATAD2 | 1.71 | Down |  |  |  |
| Glia maturation factor, beta | GMFB | 1.71 | Down |  |  |  |
| CGG triplet repeat binding protein 1 | CGGBP1 | 1.7 | Down |  |  |  |
| Muskelin 1, intracellular mediator containing kelch motifs | MKLN1 | 1.7 | Down |  |  |  |
| Homo sapiens, Similar to likely ortholog of yeast ARV1, clone IMAGE:3460560, mRNA | - | 1.7 | Down |  |  |  |
| Potassium channel tetramerisation domain containing 3 | KCTD3 | 1.7 | Down |  |  |  |
| Coiled-coil domain containing 6 | CCDC6 | 1.7 | Down |  |  |  |
| Dermatan sulfate epimerase | DSE | 1.69 | Down |  |  |  |
| RAD21 homolog (S. pombe) | RAD21 | 1.69 | Down |  |  |  |
| Dedicator of cytokinesis 7 | DOCK7 | 1.69 | Down |  |  |  |
| BCL2-associated transcription factor 1 | BCLAF1 | 1.68 | Down |  |  |  |
| Forkhead box N2 | FOXN2 | 1.68 | Down |  |  |  |
| Catenin (cadherin-associated protein), alpha-like 1 | CTNNAL1 | 1.68 | Down |  |  |  |
| ATP-binding cassette, sub-family E (OABP), member 1 | ABCE1 | 1.68 | Down |  |  |  |
| Echinoderm microtubule associated protein like 4 | EML4 | 1.68 | Down |  |  |  |
| Metadherin | MTDH | 1.68 | Down |  |  |  |
| Heat shock 105kDa/110kDa protein 1 | HSPH1 | 1.67 | Down |  |  |  |
| **Gene Name** | **Gene ID** | **Ratio** | **Direction** |  |  |  |
| ARP2 actin-related protein 2 homolog (yeast) | ACTR2 | 1.67 | Down |  |  |  |
| ELOVL family member 6, elongation of long chain fatty acids (FEN1/Elo2, SUR4/Elo3-like, yeast) | ELOVL6 | 1.67 | Down |  |  |  |
| USO1 homolog, vesicle docking protein (yeast) | USO1 | 1.67 | Down |  |  |  |
| Regulatory factor X, 7 | RFX7 | 1.67 | Down |  |  |  |
| GC-rich promoter binding protein 1 | GPBP1 | 1.67 | Down |  |  |  |
| Adenosylmethionine decarboxylase 1 | AMD1 | 1.67 | Down |  |  |  |
| Calcium binding protein 39 | CAB39 | 1.66 | Down |  |  |  |
| Family with sequence similarity 20, member B | FAM20B | 1.66 | Down |  |  |  |
| Guanine nucleotide binding protein (G protein), alpha 13 | GNA13 | 1.65 | Down |  |  |  |
| Fermitin family homolog 2 (Drosophila) | FERMT2 | 1.65 | Down |  |  |  |
| Pumilio homolog 2 (Drosophila) | PUM2 | 1.65 | Down |  |  |  |
| AT hook containing transcription factor 1 | AHCTF1 | 1.65 | Down |  |  |  |
| Protein kinase, DNA-activated, catalytic polypeptide | PRKDC | 1.64 | Down |  |  |  |
| Amyloid beta precursor protein (cytoplasmic tail) binding protein 2 | APPBP2 | 1.64 | Down |  |  |  |
| Solute carrier family 30 (zinc transporter), member 9 | SLC30A9 | 1.64 | Down |  |  |  |
| Family with sequence similarity 178, member A | FAM178A | 1.63 | Down |  |  |  |
| Pleckstrin homology domain interacting protein | PHIP | 1.63 | Down |  |  |  |
| Solute carrier family 7 (cationic amino acid transporter, y+ system), member 2 | SLC7A2 | 1.63 | Down |  |  |  |
| Ubiquitin protein ligase E3 component n-recognin 5 | UBR5 | 1.63 | Down |  |  |  |
| PREDICTED: misc_RNA (LOC647150), miscRNA. | - | 1.62 | Down |  |  |  |
| Zinc finger homeobox 4 | ZFHX4 | 1.62 | Down |  |  |  |
| Matrin 3 | MATR3 | 1.62 | Down |  |  |  |
| CDNA FLJ12874 fis, clone NT2RP2003769 | - | 1.62 | Down |  |  |  |
| Protein tyrosine phosphatase, non-receptor type 11 | PTPN11 | 1.62 | Down |  |  |  |
| taurine upregulated 1 (non-protein coding) | - | 1.61 | Down |  |  |  |
| Leucine-rich PPR-motif containing | LRPPRC | 1.61 | Down |  |  |  |
| CKLF-like MARVEL transmembrane domain containing 6 | CMTM6 | 1.61 | Down |  |  |  |
| KIAA0528 | KIAA0528 | 1.61 | Down |  |  |  |
| Armadillo repeat containing, X-linked 3 | ARMCX3 | 1.6 | Down |  |  |  |
| DCN1, defective in cullin neddylation 1, domain containing 4 (S. cerevisiae) | DCUN1D4 | 1.6 | Down |  |  |  |
| Rho GTPase activating protein 21 | ARHGAP21 | 1.59 | Down |  |  |  |
| Microtubule-actin crosslinking factor 1 | MACF1 | 1.59 | Down |  |  |  |
| Calmodulin regulated spectrin-associated protein 1-like 1 | CAMSAP1L1 | 1.59 | Down |  |  |  |
| HECT domain containing 1 | HECTD1 | 1.59 | Down |  |  |  |
| Discs, large (Drosophila) homolog-associated protein 5 | DLGAP5 | 1.59 | Down |  |  |  |
| Pericentriolar material 1 | PCM1 | 1.59 | Down |  |  |  |
| Stromal antigen 2 | STAG2 | 1.59 | Down |  |  |  |
| Regulatory solute carrier protein, family 1, member 1 | RSC1A1 | 1.59 | Down |  |  |  |
| Polymerase (DNA directed), alpha 1, catalytic subunit | POLA1 | 1.59 | Down |  |  |  |
| UPF2 regulator of nonsense transcripts homolog (yeast) | UPF2 | 1.59 | Down |  |  |  |
| F-box protein 11 | FBXO11 | 1.58 | Down |  |  |  |
| Golgi autoantigen, golgin subfamily a, 8A | GOLGA8A | 1.58 | Down |  |  |  |
| Methionine sulfoxide reductase B3 | MSRB3 | 1.58 | Down |  |  |  |
| P21 protein (Cdc42/Rac)-activated kinase 2 | PAK2 | 1.58 | Down |  |  |  |
| **Gene Name** | **Gene ID** | **Ratio** | **Direction** |  |  |  |
| Pericentriolar material 1 | PCM1 | 1.58 | Down |  |  |  |
| Topoisomerase (DNA) II beta 180kDa | TOP2B | 1.58 | Down |  |  |  |
| NOP58 ribonucleoprotein homolog (yeast) | NOP58 | 1.58 | Down |  |  |  |
| DEAD (Asp-Glu-Ala-Asp) box polypeptide 18 | DDX18 | 1.58 | Down |  |  |  |
| Zinc finger protein 146 | ZNF146 | 1.57 | Down |  |  |  |
| Heat shock protein 90kDa alpha (cytosolic), class A member 1 | HSP90AA1 | 1.57 | Down |  |  |  |
| Septin 11 | 11-Sep | 1.57 | Down |  |  |  |
| Discs, large (Drosophila) homolog-associated protein 5 | DLGAP5 | 1.57 | Down |  |  |  |
| Additional sex combs like 2 (Drosophila) | ASXL2 | 1.57 | Down |  |  |  |
| RNA binding motif protein 12 | RBM12 | 1.57 | Down |  |  |  |
| SERPINE1 mRNA binding protein 1 | SERBP1 | 1.57 | Down |  |  |  |
| Discoidin, CUB and LCCL domain containing 2 | DCBLD2 | 1.57 | Down |  |  |  |
| Activated leukocyte cell adhesion molecule | ALCAM | 1.56 | Down |  |  |  |
| Zinc finger, BED-type containing 5 | ZBED5 | 1.56 | Down |  |  |  |
| SMAD family member 5 | SMAD5 | 1.56 | Down |  |  |  |
| Kinesin family member 23 | KIF23 | 1.56 | Down |  |  |  |
| Forty-two-three domain containing 1 | FYTTD1 | 1.56 | Down |  |  |  |
| EH domain binding protein 1 | EHBP1 | 1.56 | Down |  |  |  |
| Latrophilin 2 | LPHN2 | 1.56 | Down |  |  |  |
| Nudix (nucleoside diphosphate linked moiety X)-type motif 21 | NUDT21 | 1.56 | Down |  |  |  |
| La ribonucleoprotein domain family, member 4 | LARP4 | 1.56 | Down |  |  |  |
| Carboxypeptidase D | CPD | 1.56 | Down |  |  |  |
| RAP2A, member of RAS oncogene family | RAP2A | 1.55 | Down |  |  |  |
| Heat shock protein 90kDa alpha (cytosolic), class A member 1 | HSP90AA1 | 1.55 | Down |  |  |  |
| Caldesmon 1 | CALD1 | 1.55 | Down |  |  |  |
| Kinesin family member 11 | KIF11 | 1.55 | Down |  |  |  |
| PREDICTED: similar to protein kinase, DNA-activated, catalytic polypeptide (LOC731751), mRNA. | - | 1.55 | Down |  |  |  |
| Exportin 4 | XPO4 | 1.55 | Down |  |  |  |
| Centrosomal protein 55kDa | CEP55 | 1.55 | Down |  |  |  |
| Heat shock 70kDa protein 4-like | HSPA4L | 1.54 | Down |  |  |  |
| Dpy-19-like 1 (C. elegans) | DPY19L1 | 1.54 | Down |  |  |  |
| ELOVL family member 6, elongation of long chain fatty acids (FEN1/Elo2, SUR4/Elo3-like, yeast) | ELOVL6 | 1.54 | Down |  |  |  |
| Membrane-associated ring finger (C3HC4) 7 | 7-Mar | 1.54 | Down |  |  |  |
| UBX domain protein 4 | UBXN4 | 1.54 | Down |  |  |  |
| Chromodomain helicase DNA binding protein 9 | CHD9 | 1.53 | Down |  |  |  |
| TROVE domain family, member 2 | TROVE2 | 1.53 | Down |  |  |  |
| Striatin, calmodulin binding protein 3 | STRN3 | 1.53 | Down |  |  |  |
| Zinc finger, RAN-binding domain containing 2 | ZRANB2 | 1.53 | Down |  |  |  |
| Glutamyl-prolyl-tRNA synthetase | EPRS | 1.53 | Down |  |  |  |
| LAG1 homolog, ceramide synthase 6 | LASS6 | 1.53 | Down |  |  |  |
| SDA1 domain containing 1 | SDAD1 | 1.53 | Down |  |  |  |
| Ribosomal protein S6 kinase, 70kDa, polypeptide 1 | RPS6KB1 | 1.53 | Down |  |  |  |
| DEAH (Asp-Glu-Ala-His) box polypeptide 29 | DHX29 | 1.53 | Down |  |  |  |
| Ubiquitin protein ligase E3 component n-recognin 5 | UBR5 | 1.52 | Down |  |  |  |
| Zinc finger and BTB domain containing 33 | ZBTB33 | 1.52 | Down |  |  |  |
| Non imprinted in Prader-Willi/Angelman syndrome 1 | NIPA1 | 1.52 | Down |  |  |  |
| SMG1 homolog, phosphatidylinositol 3-kinase-related kinase (C. elegans) | SMG1 | 1.52 | Down |  |  |  |
| **Gene Name** | **Gene ID** | **Ratio** | **Direction** |  |  |  |
| Matrin 3 | MATR3 | 1.52 | Down |  |  |  |
| SWI/SNF related, matrix associated, actin dependent regulator of chromatin, subfamily a, member 5 | SMARCA5 | 1.52 | Down |  |  |  |
| REST corepressor 3 | RCOR3 | 1.52 | Down |  |  |  |
| NMD3 homolog (S. cerevisiae) | NMD3 | 1.52 | Down |  |  |  |
| Platelet-activating factor acetylhydrolase, isoform Ib, subunit 1 (45kDa) | PAFAH1B1 | 1.52 | Down |  |  |  |
| Kelch domain containing 5 | KLHDC5 | 1.52 | Down |  |  |  |
| Kinetochore associated 1 | KNTC1 | 1.52 | Down |  |  |  |
| Kinesin family member 11 | KIF11 | 1.51 | Down |  |  |  |
| Aminoadipate-semialdehyde dehydrogenase-phosphopantetheinyl transferase | AASDHPPT | 1.51 | Down |  |  |  |
| RAS p21 protein activator (GTPase activating protein) 1 | RASA1 | 1.5 | Down |  |  |  |
| Tetratricopeptide repeat domain 3 | TTC3 | 1.5 | Down |  |  |  |
| Cysteine and histidine-rich domain (CHORD)-containing 1 | CHORDC1 | 1.5 | Down |  |  |  |
| THO complex 2 | THOC2 | 1.5 | Down |  |  |  |
| Zinc finger protein 770 | ZNF770 | 1.5 | Down |  |  |  |
| CD46 molecule, complement regulatory protein | CD46 | 1.5 | Down |  |  |  |
| Transmembrane protein 181 | TMEM181 | 1.5 | Down |  |  |  |
