# Supplementary material for: Trans10,cis12 conjugated linoleic acid inhibits proliferation and migration of ovarian cancer cells by inducing ER stress, autophagy, and modulation of Src
Source: PLoS One. 2018 Jan 11;13(1):e0189524. doi: 10.1371/journal.pone.0189524 (PMC5764254; doi:10.1371/journal.pone.0189524)
Supplement: S2 Table — Z-score is the measure of standard deviation. A Z-score of 2 indicates that the value is two standard deviations away from mean. (DOCX) [file pone.0189524.s002.docx]

**S2 Table**

|  |  |  |  |  |  |  |
| --- | --- | --- | --- | --- | --- | --- |
| KEGG Pathway | List | Up | Down | Gene Set | z-score (Up) | z-score (Down) |
| Protein processing in endoplasmic reticulum | 7 | 4 | 3 | 163 | 7.07 | 1.73 |
| MAPK signaling pathway | 5 | 3 | 2 | 267 | 3.8 | 0.06 |
| Regulation of actin cytoskeleton | 5 | 1 | 4 | 208 | 1.08 | 2.1 |
| mRNA surveillance pathway | 4 | 0 | 4 | 82 | -0.38 | 4.5 |
| RNA transport | 4 | 0 | 4 | 153 | -0.52 | 2.82 |
| Vascular smooth muscle contraction | 4 | 1 | 3 | 123 | 1.71 | 2.29 |
| Cell cycle | 3 | 0 | 3 | 123 | -0.47 | 2.29 |
| ErbB signaling pathway | 3 | 1 | 2 | 86 | 2.21 | 1.78 |
| Long-term potentiation | 3 | 2 | 1 | 70 | 5.41 | 0.71 |
| Neurotrophin signaling pathway | 3 | 2 | 1 | 127 | 3.82 | 0.1 |
| Prostate cancer | 3 | 2 | 1 | 88 | 4.75 | 0.47 |
| Renal cell carcinoma | 3 | 1 | 2 | 70 | 2.53 | 2.14 |
| Spliceosome | 3 | 0 | 3 | 125 | -0.47 | 2.26 |
| Acute myeloid leukemia | 2 | 1 | 1 | 56 | 2.9 | 0.95 |
| Chronic myeloid leukemia | 2 | 1 | 1 | 72 | 2.48 | 0.68 |
| GnRH signaling pathway | 2 | 2 | 0 | 98 | 4.46 | -0.85 |
| Long-term depression | 2 | 1 | 1 | 68 | 2.57 | 0.74 |
| mTOR signaling pathway | 2 | 0 | 2 | 51 | -0.3 | 2.73 |
| Porphyrin and chlorophyll metabolism | 2 | 1 | 1 | 32 | 4.01 | 1.62 |
| Thyroid cancer | 2 | 1 | 1 | 28 | 4.31 | 1.8 |
| B cell receptor signaling pathway | 1 | 1 | 0 | 75 | 2.42 | -0.74 |
| Biosynthesis of unsaturated fatty acids | 1 | 0 | 1 | 21 | -0.19 | 2.2 |
| Bladder cancer | 1 | 1 | 0 | 41 | 3.48 | -0.55 |
| Endometrial cancer | 1 | 1 | 0 | 51 | 3.07 | -0.61 |
| Fc epsilon RI signaling pathway | 1 | 1 | 0 | 77 | 2.38 | -0.75 |
| Gap junction | 1 | 1 | 0 | 89 | 2.16 | -0.81 |
| Glioma | 1 | 1 | 0 | 65 | 2.65 | -0.69 |
| Glycerophospholipid metabolism | 1 | 1 | 0 | 77 | 2.38 | -0.75 |
| Glycosaminoglycan biosynthesis - chondroitin sulfate | 1 | 0 | 1 | 22 | -0.2 | 2.13 |
| Melanoma | 1 | 1 | 0 | 71 | 2.5 | -0.72 |
| Mismatch repair | 1 | 0 | 1 | 22 | -0.2 | 2.13 |
| Non-homologous end-joining | 1 | 0 | 1 | 13 | -0.15 | 2.98 |
| Non-small cell lung cancer | 1 | 1 | 0 | 54 | 2.96 | -0.63 |
| Prion diseases | 1 | 1 | 0 | 36 | 3.75 | -0.51 |
| Terpenoid backbone biosynthesis | 1 | 0 | 1 | 15 | -0.16 | 2.74 |
| VEGF signaling pathway | 1 | 1 | 0 | 73 | 2.46 | -0.73 |
