# Supplementary figures and images for: Trans10,cis12 conjugated linoleic acid inhibits proliferation and migration of ovarian cancer cells by inducing ER stress, autophagy, and modulation of Src
Source: PLoS One. 2018 Jan 11;13(1):e0189524. doi: 10.1371/journal.pone.0189524 (PMC5764254; doi:10.1371/journal.pone.0189524)

S1 Fig.

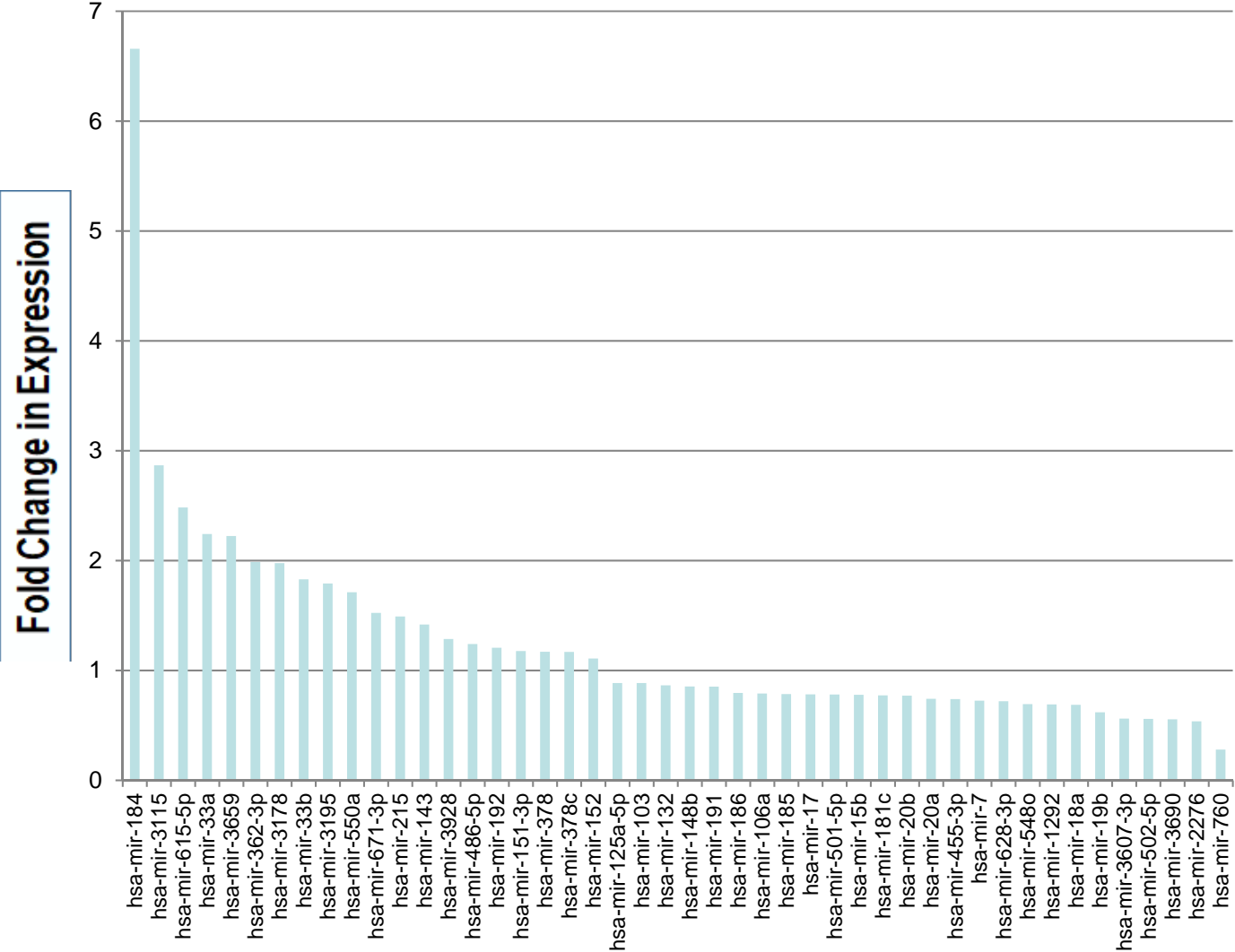

Supplement: S1 Fig — (PDF) [file pone.0189524.s003.pdf]
